# A Nerve Cell Growth Promoting PEG-Peptide Block Copolymer and Photoresponsive Hydrogels with Tailorable Mechanical Properties and Feasible Degradability

**DOI:** 10.1021/acspolymersau.5c00165

**Published:** 2026-01-20

**Authors:** Syuan-Yu Lin, Wei-Fang Su, Chun-Yu Chang, Chi-Yang Chao

**Affiliations:** † Department of Materials Science and Engineering, 33561 National Taiwan University, No. 1, Sec. 4, Roosevelt Road, Taipei 10617, Taiwan; ‡ Department of Materials Engineering, Ming-Chi University of Technology, 84 Gungjuan Rd., Taishan Dist., New Taipei City 243303, Taiwan; § Bachelor Program in Semiconductor Materials and Fabrication, Ming Chi University of Technology, 84 Gungjuan Rd., Taishan Dist., New Taipei City 243303, Taiwan; ∥ Biochemical Technology R&D Center, Ming Chi University of Technology, 84 Gungjuan Rd., Taishan Dist., New Taipei City 243303, Taiwan; ⊥ Advanced Research Center for Green Materials Science and Technology, 33561 National Taiwan University, No. 1, Sec. 4, Roosevelt Road, Taipei 10617, Taiwan

**Keywords:** hydrogel, PEG-pepitde, coumarin, tissue engineering, bridge-micelle architecture

## Abstract

In this study, a novel photoresponsive poly­(ethylene glycol)-peptide (PEG-peptide) diblock copolymer capable of promoting pheochromocytoma cell (PC12) growth is developed, and the corresponding hydrogels with tunable mechanical properties for nerve tissue engineering are constructed via bridge-micelle architectures. The PEG-peptide forms core–shell micelles in the precursor solution, in which the core peptide segment contains γ-benzyl-l-glutamate moieties to stimulate nerve cell growth and coumarin moieties to provide photoresponsivity, while the hydrophilic PEG shell could enhance stable dispersion of micelles. Meanwhile, coumarin-containing water-soluble random copolymers poly­(*N*,*N*-dimethylacrylamide-random-acrylic­(7-(2-acryloyloxyethoxy)-4-methylcoumarin)) (PDA) are incorporated to function as bridges. The coumarin moieties in both polymers undergo [2 + 2] cycloaddition upon 365 nm UV irradiation, resulting in the coexistence of three different types of cross-linking: intramicelle, micelle-bridge, and interbridge cross-linking. By adjusting the composition and concentration of the precursor solutions as well as 365 nm UV irradiation time to delicately balance these cross-linkings, hydrogels with a wide range of mechanical strengths, swelling ratios, and viscoelastic behaviors are feasibly fabricated. This construction not only expands the gelation window but also exerts an effective approach to precisely modulate mechanical properties and water absorption of hydrogels, which could further optimize the environment for cell growth. The complex modulus of the hydrogels is tunable between 238 and 1448 Pa, aligned with the mechanical strength of native extracellular matrix for PC12 cell growth. It is noteworthy that a high complex modulus and high swelling ratio could be concurrently achieved, enabling excellent PC12 cell growth performance in cell cytotoxicity and 3.2 times cell viability with respect to the control group. Additionally, upon 30 min of 254 nm UV irradiation, the hydrogels can be un-cross-linked into solutions via dedimerization of coumarin, offering a great potential for clean scaffold removal. These achievements demonstrate that the hydrogel system provides a cytocompatible and supportive biochemical environment, offering promising potential as a foundational platform for nerve-regeneration scaffold design.

## Graphical abstract

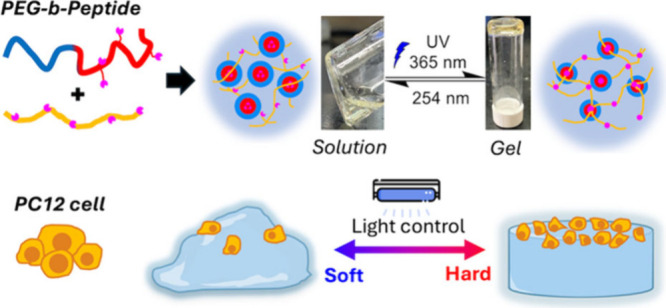

## Introduction

Nerve injuries pose significant challenges in the medical and healthcare fields, representing one of the leading causes of disability worldwide. The resulting motor and sensory deficits, chronic pain, and reduced quality of life demonstrate the urgent need for effective treatment strategies to promote nerve repair and functionality recovery.^1,2^ Nerve tissue engineering has emerged as an innovative approach for restoring neural function.^3–6^ A core aspect is to construct biocompatible scaffolds with nerve regeneration promoters to serve as substrates for cell growth, differentiation, and maintenance of biological function.^7–10^ The chemical structure and mechanical properties of scaffolds play important roles in regulating cellular behaviors, such as migration, adhesion, and differentiation. Therefore, precisely modulating the physical properties of scaffolds, including stiffness and viscoelasticity, is key to optimize extracellular matrix (ECM) environment for different cells.^11–14^

Hydrogels have high water content and excellent biocompatibility, allowing them to closely mimic native ECM environment as ideal substrates for biological scaffolds.^15–17^ Conventional hydrogels are typically prepared through either physical or chemical cross-linking of polymers in an aqueous solution, which generally exhibited limited gelation windows and narrow ranges of mechanical properties. In recent years, nanocomposite hydrogels incorporating self-assembled micelles as cross-linkers or fillers have gained increasing attention due to their potential to extend mechanical properties and encapsulate therapeutic agents.^18–20^ Another eye-catching hydrogel design strategy is incorporating reversible photodimerization groups, such as anthracene, coumarin, and diarylethene, to offer the hydrogels photostimuli responsivities.^21–24^ Among these candidates, coumarin derivatives are of special interests because of their nature existence and good biocompatibility.^23,24^ Coumarin undergoes effective [2 + 2] cycloaddition upon 365 nm UV irradiation to form covalently bonded dimers, allowing dynamic and spatiotemporally controlled cross-linking to modulate the mechanical properties of the hydrogels. Meanwhile, the dimerized moieties could be disengaged when treated with 254 nm irradiation,^25–27^ enabling the hydrogel return to a solution for feasible scaffold removal without the need of enzymatic or chemical degradation agents, reducing potential cytotoxicity and immune responses.

Previous research has demonstrated poly­(γ-benzyl-l-glutamate) (PBG) based scaffolds exhibit good biocompatibility and ability to promote nerve cell growths.^5–8^ These studies adopted electrospun fibrils as the supporting materials for the scaffolds, which exhibited a much higher rigidity compared to native tissues. In this work, we aim to develop photoresponsive hydrogels for applications in nerve regeneration scaffolds with inherent softness mimicking the native ECM environments. These hydrogels are also anticipated to exhibit superior properties including tailorable mechanical properties and photolysis capability by combining advantages of micelle-containing nanocomposites and reversible photoresponsivity of coumarin. Accordingly, we design and synthesize novel coumarin-functionalized PEG-peptide block copolymers (BCP) whose structure and synthesis routes are shown in Figure 1. The BCP consists of a hydrophilic poly­(ethylene glycol) (PEG) segment as well as a hydrophobic peptide segment containing γ-benzyl-l-glutamate (BG), glutaric acid (GA), and coumarin (C) groups. The peptide segment is a random copolymer, in which BG is to promote nerve cell growths, GA is to modulate the hydrophobicity, and C is to provide reversible photoresponsivity. The PEG segment is covalently linked to the peptide segment in order to self-assemble into water-dispersible core–shell micelles. To construct a hydrogel, a poly­(*N*,*N*-dimethylacrylamide-r-acrylic­(7-(2-acryloyloxyethoxy)-4-methylcoumarin)) (PDA) random copolymer is incorporated to act as bridges to form a bridge-micelle network for the structural foundation. Upon irradiation with 365 nm UV light, the coumarin moieties in both PDA and PEG-peptide BCPs undergo photodimerization to form cross-links and thus stable hydrogels. Meanwhile, the dimerized coumarin could be disengaged upon 254 nm UV irradiation, allowing the hydrogel to proceed un-cross-linking and retrieve the solution status.

**Figure 1:**
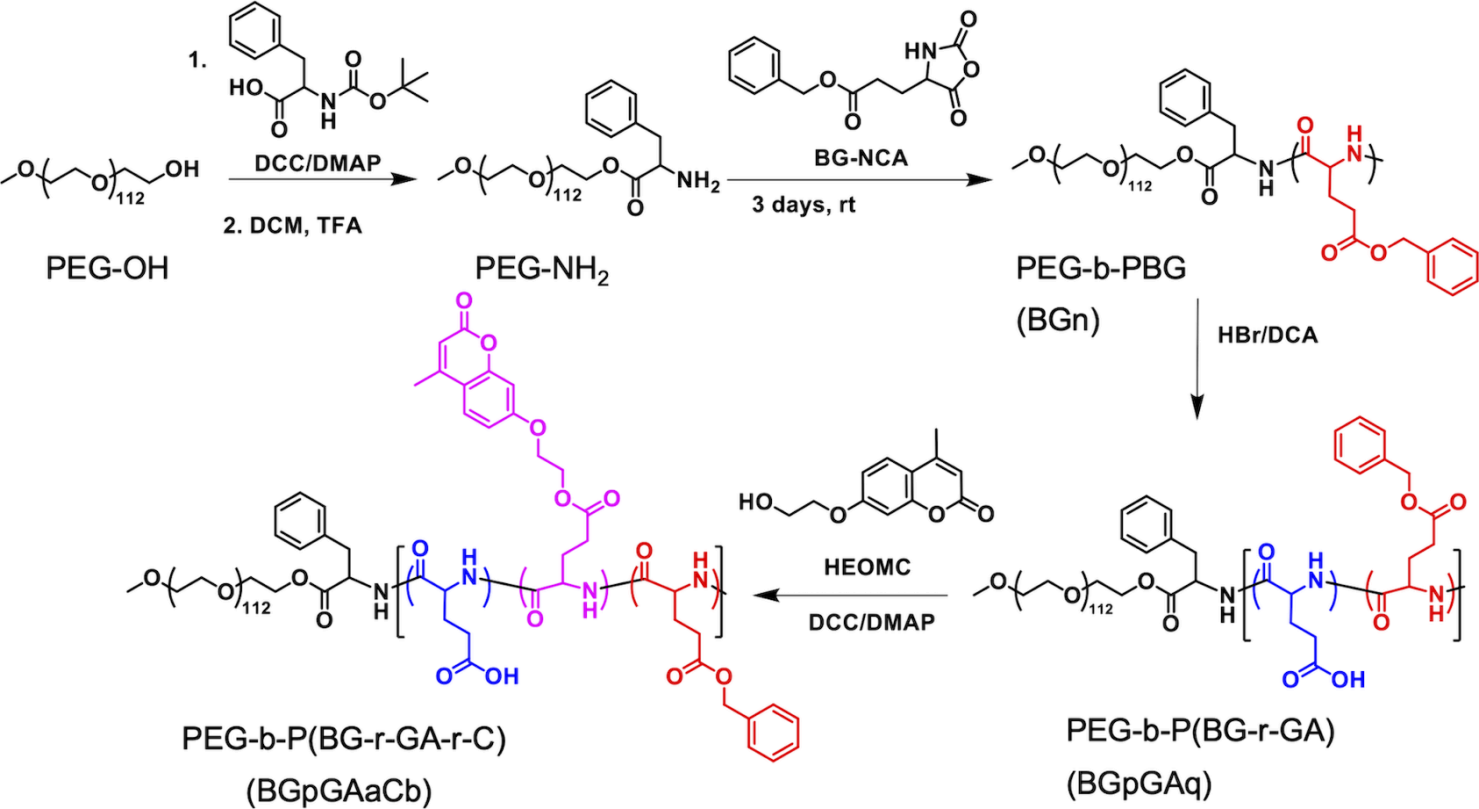
Synthetic route of PEG-*b*-P­(BG-r-GA-r-C).

To successfully produce hydrogels, homogeneous aqueous micellar dispersions in high concentration are necessary, which is achieved by carefully tailoring the composition of the block copolymer to balance the hydrophilicity/hydrophobicity between PEG/peptide segments. Afterward, a series of precursor aqueous solutions comprising PDA and PEG-peptide BCP with different compositions and concentrations are prepared and subjected to UV irradiation. By adjusting the precursor solution and the UV treatment duration, we can feasibly modulate the mechanical properties, water absorption capability, and viscoelastic behaviors of the hydrogels in a wide range. The PC12 cellular activities are evaluated including cell cytotoxicity using a live/dead assay and cell viability using an Alamar Blue assay, which are found to closely relate to the mechanical strengths and BG contents of the hydrogels. This tunability enables the creation of diverse microenvironments to accommodate specific biological requirements for different cells without the need for tremendous works on synthesizing a variety of polymers having different compositions and chemical structures. Photo dedimerization studies of the hydrogels are also performed to demonstrate the potential of safe and clean removal of the scaffold. We believe that this work should contribute to the advancement of next-generation nerve regeneration scaffolds, with promising implications for broader clinical applications.

## Results and Discussion

### Design and Synthesis of BG15GA10C5 Block Copolymers

The synthesis route of PEG-peptide block copolymers is illustrated in Figure 1. The macroinitiator PEG-NH_2_, an amine-terminated poly­(ethylene glycol) with a degree of polymerization (DPn) of 112, is synthesized from PEG–OH according to a previously reported method and serves as a hydrophilic segment in the block copolymer.^28^ The terminal amine group readily undergoes ring-opening polymerization of γ-benzyl-l-glutamate-*N*-carboxyanhydride (BG-NCA) for the polypeptide segment with neuron-regenerative capability, yielding a PEG-*b*-PBG diblock copolymer, denoted as BG*n* where *n* is the number of BG units in PBG. Since the phenyl ring of BG units makes PBG inherently hydrophobic, PEG-*b*-PBG exhibits limited solubility and poor dispersibility in water. To improve the hydrophilicity of the PBG block, PEG-*b*-PBG is treated with an acidic HBr aqueous solution to partially hydrolyze the BG unit into l-glutamic acid (GA) by selectively cleaving the ester bonds, affording water-dispersible PEG-*b*-P­(BG-r-GA) diblock copolymer, termed BG*p*GA*q*, in which *p* is the number of BG units and *q* is the number of GA units. To impart reversible and tunable photo-cross-linking properties, coumarin-functionalized moieties 7-(2-hydroxyethoxy)-4-methyl-2H-chromen-2-one (HEOMC) are grafted onto the GA units through esterification using DCC/DMAP as the catalyst to produce the target PEG-peptide block copolymer PEG-*b*-P­(BG-r-GA-r-C), denoted as BG*p*GA*a*C*b*, where *a* is the number of GA units after modification and *b* is the number of coumarin grafted GA units. Table 1 summarizes the compositions and the molecular weight related information on all precursor polymers (BG*n* and BG*p*GA*q*) and the target polymer (BG*p*GA*a*C*b*).

**Table 1:** Structural Composition of the PEG-Peptide Polymers

		composition (from ^1^H NMR)			molecule weight (from GPC)		
sample	structure	number of BG	number of GA	number of C	*M*_n_	*M*_w_	PDI
BG30	PEG-*b*-PBG	30	–	–	14,500	17,000	1.17
BG25GA5	PEG-*b*-P(BG-r-GA)	25	5	–	12,400	16,370	1.32
BG21GA9		21	9	–	11,600	13,790	1.18
BG15GA15		15	15	–	9830	11,360	1.15
BG15GA10C5	PEG-*b*-P(BG-r-GA-r-C)	15	10	5	13,430	18,710	1.39

Figure 2(a) depicts the ^1^H NMR spectrum of PEG-NH_2_. The ratio between the integral of the terminal methoxy signal of PEG at δ = 3.26 ppm (H_a_) and that of the terminal aromatic protons at δ = 7.2–7.42 ppm (H_b_) is 3/4.98, very close the theoretical ratio of H_a_/H_b_ at 3/5, which suggests the complete conversion of PEG–OH to PEG–NH_2_. The number of BG units (*n*) in PEG-*b*-PBG is determined to be 30 from the ^1^H NMR spectrum in Figure 2(b) by comparing the integral of the terminal methoxy signal of PEG (H_a_) with that of the aromatic proton signals of BG (H_c_). Detailed calculations are provided in the Supporting Information (Figure S11). The corresponding molecular weight of the PBG block is calculated to be approximately 6600, yielding a total number-average molecular weight (*M*_n_) of 11,600 when combined with the 5000 Da PEG segment. This value is in reasonable agreement with the GPC-determined *M*_n_ of 14,500. Moreover, the polydispersity index (PDI) obtained from GPC remains below 1.2, indicating a narrow molecular weight distribution of the PBG segment.

**Figure 2:**
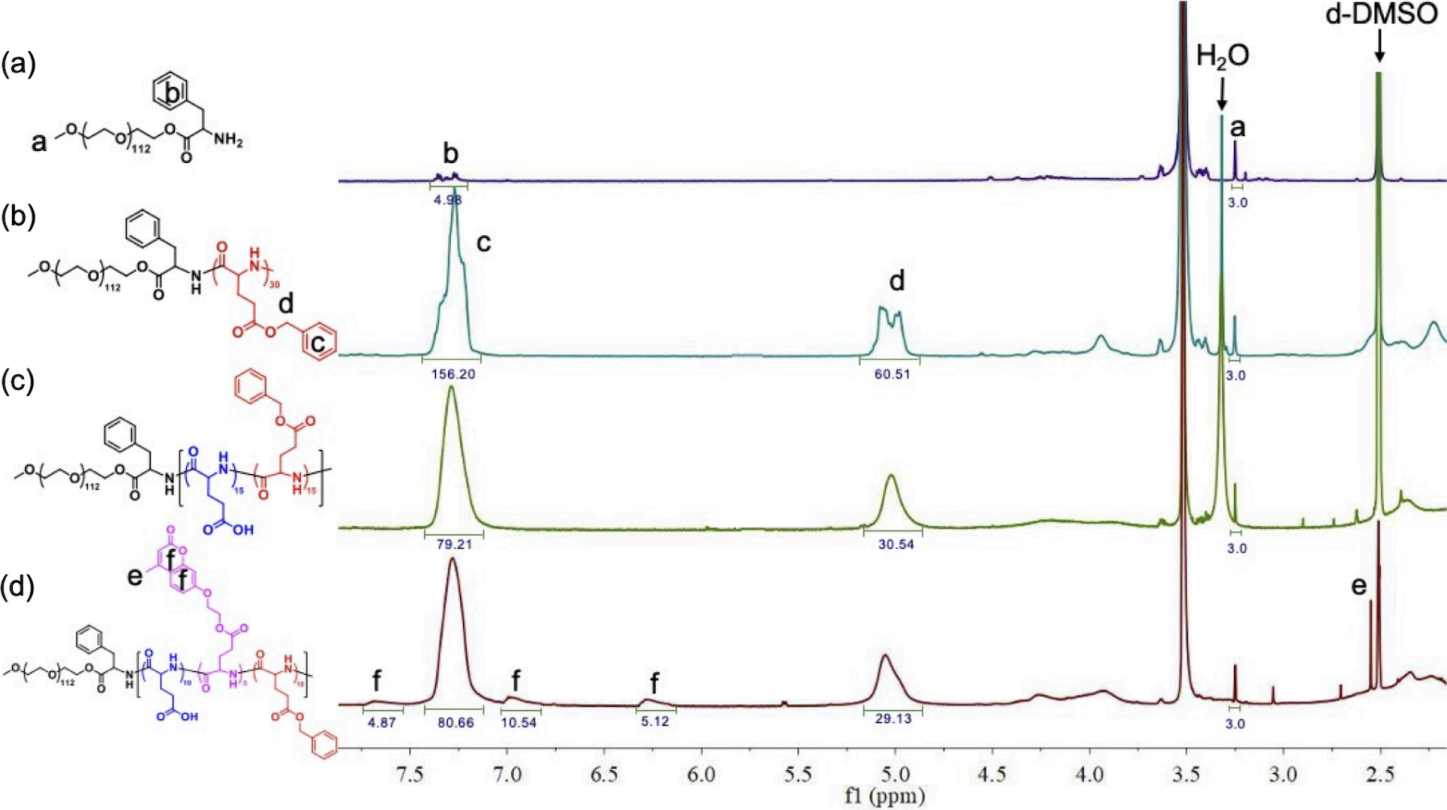
^1^H NMR spectra of (a) PEG-NH_2_, (b) BG30, (c) BG15GA15, and (d) BG15GA10C5.

BG*n* is then subjected to partial hydrolysis to afford BG*p*GA*q*, in which part of the BG units are converted to GA. The degree of hydrolysis, defined as the number of GA units (*q*) over the number of BG units (*n*) in BG*n*, is adjusted by varying the concentration of HBr and is identified by ^1^H NMR spectroscopy. Taking BG15GA15 as an example, whose ^1^H NMR spectrum is shown in Figure 2(c), the integral of the peaks at δ = 7.2–7.42 ppm associated with the phenyl groups of BG (H_c_) is clearly reduced to suggest successful hydrolysis of PBG. The degree of hydrolysis is calculated based on the difference of H_c_ integral between BG*p*GA*q* and BG*n*. Three BG*p*GA*q* with 50%, 30%, and 18% degree of hydrolysis are prepared, and the corresponding numbers of BG units (*p*) and GA units (*q*) are listed in Table 1. The associated calculations are detailed in the Supporting Information (Figure S11). As seen in Figure 3(a), the degree of hydrolysis increases with an increasing concentration of HBr in a linear fashion. The GPC results of the three BG*p*GA*q* suggest a lower molecular weight compared to BG30, and the molecular weight decreases with increasing degree of hydrolysis, i.e., decreasing *p* in BG*p*GA*q*, which is in accordance with the ^1^H NMR results. Additionally, the *M*_n_ obtained from ^1^H NMR is in good agreement with *M*_n_ retrieved from GPC. Taking BG15GA15 for example, *M*_n_ estimated from ^1^H NMR is 10,500, which closely matches the GPC-determined *M*_n_ of 9830, confirming the accuracy of the composition of BG*p*GA*q*. Furthermore, the PDI of the three BG*p*GA*q* still remains smaller than 1.4. These observations confirm the success of controlled hydrolysis without side reactions such as main chain degradation and/or cross-linking.

**Figure 3:**
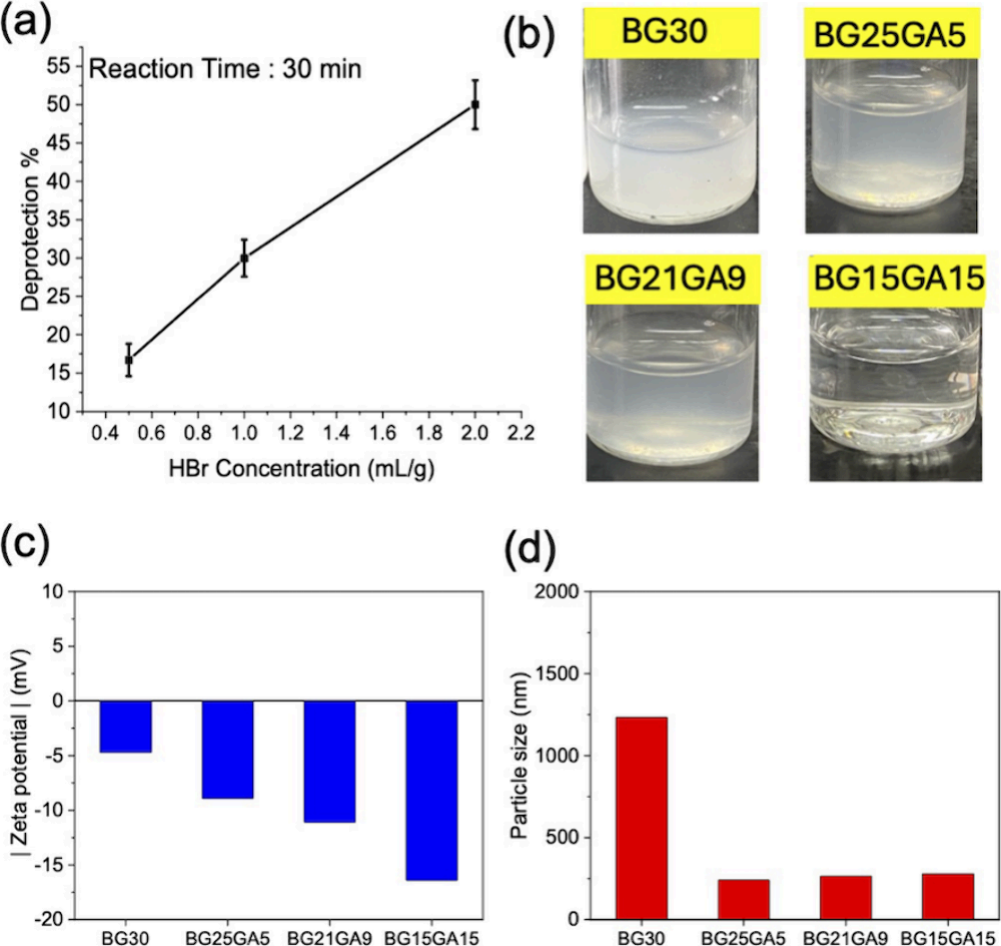
(a) The relationship between HBr concentration and degree of hydrolysis of BGpGAq; (b) appearance of 1 wt % BGpGAq aqueous solutions; (c) zeta potential of 0.1 wt % BGpGAq solution, and (d) particle size of 0.1 wt % BGpGAq polymer solution from DLS analysis.

Although increasing the degree of hydrolysis should improve the water solubility of the P­(BG-r-GA) block; the reduction in BG moieties would also limit the nerve regeneration capability. It is important to find the optimal composition of P­(BG-r-GA) segment in BG*p*GA*q* to allow the target PEG-peptide block copolymer possessing enough water dispersity for hydrogel formation while retaining good capability for nerve regeneration. Figure 3(b) shows the photos of 1 wt % aqueous solution of BG30 and BG*p*GA*q*, in which BG30 appears very cloudy and the solubility of BG*p*GA*q* improved progressively as the GA content increased. We also investigated the stability of the aqueous dispersion of three BG*p*GA*q* through zeta potential measurements (Figure 3(c)). Consistent with the visual observations, the absolute zeta potential increases with increasing GA content. Specifically, BG15GA15 formed a clear and transparent solution whose zeta potential is the highest, indicating BG15GA15 has the best colloidal stability in the aqueous solution. Dynamic light scattering analysis of the aqueous solutions suggest BG30 exhibits a much larger particle size of 700 nm because of poor water solubility. By contrast, all three BG*p*GA*q* form homogeneous dispersions of colloidal particles averaging ∼200 nm in diameter (Figure 3(d)). Although the particle size distributions of BG21GA9 and BG25GA5 are similar to that of BG15GA15, both copolymers still exhibited visible turbidity in 1 wt % aqueous solutions attributed to their relatively lower zeta potential. These observations suggest that increasing GA content should effectively reduce particle size and mitigate aggregation of PEG-*b*-P­(BG-r-GA) in water, thus forming stable colloidal dispersions. Since the P­(BG-r-GA) segment needs to be further decorated with hydrophobic coumarin to obtain the target PEG-*b*-P­(BG-r-GA-r-C), to ensure it has adequate solubility for hydrogel formation, BG15GA15 showing the best water solubility is adopted for the further modification.

The content of coumarin in PEG-*b*-P­(BG-r-GA-r-C) is determined from the corresponding ^1^H NMR spectrum in Figure 2(d), in which the presence of the characteristic signals of the coumarin groups H_f_ clearly suggests the successful attachment of coumarin onto P­(BG-r-GA). The integral of H_f_ is used to quantify the number of coumarin groups in the copolymer, and the detailed calculations are provided in the Supporting Information (Figure S12). An average of one-third of the GA units are grafted with coumarin, and the resulting copolymer is termed as BG15GA10C5. Based on the ^1^H NMR spectrum, the coumarin content of BG15GA10C5, defined as the weight percentage of coumarin in per unit weight of the polymer, is determined to be 9.6 wt % as detailed in the Supporting Information (Figure S12), which closely agrees with the value of obtained from. The content of coumarin could be also obtained from eq 1 using the UV–vis absorbance at 320 nm of a 0.1 wt % BG15GA15C5 aqueous solution (Figure 4(a)), which is calculated to be 9.2 wt %, in good agreement with ^1^H NMR results. The details are provided in the Materials and Methods. The 1 wt % BG15GA10C5 solution also exhibits strong fluorescence after UV irradiation, as shown in Figure 4(b). GPC results of BG15GA10C5 show an apparent increase in molecular weight compared to BG15GA15. The molecular weight calculated from ^1^H NMR integration was 11,450, reasonably close to the GPC-measured value of 13,420, further validating the successful synthesis of the coumarin-functionalized PEG-peptide copolymer.

1 $$\mathrm{coumarin content}\;\%=\frac{A\times M}{\left[P\right]\times \varepsilon \times b}$$

in which *A* represents the absorbance at 320 nm, *M* represents the molecular weight of the HEOMC (220.22 Da), [*P*] is the weight of the target polymer per milliliter of solution, ε is the molar absorptivity (14,500 M^–1^ cm^–1^) of coumarin, and *b* is the optical path length.

**Figure 4:**
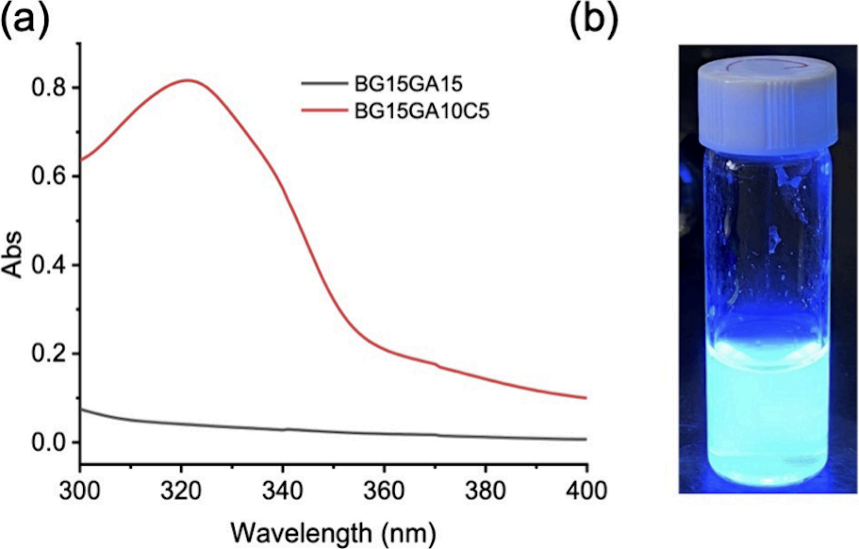
(a) UV–vis spectra of 0.1 wt % BG15GA15 and BG15GA10C5 aqueous solutions; (b) the photo of 1 wt % BG15GA10C5 aqueous solution upon 365 nm UV irradiation.

### Photoresponsivity of Coumarin-Functionalized PEG-Peptide

The photodimerization behavior of BG15GA10C5 in a 0.15 wt % aqueous solution under UV irradiation at 365 nm is evaluated according to the time-dependent UV absorption spectra in Figure 5(a). A gradual decrease in the absorbance at 320 nm, corresponding to the characteristic absorbance of coumarin, over exposure time is observed, indicating the degree of cross-linking increases with the exposure time. Afterward, the photo-cross-linked BG15GA10C5 was exposed to 254 nm UV radiation, and the absorbance at 320 nm progressively increases (Figure 5(b)), signifying the occurrence of dedimerization to retrieve unreacted coumarin. As seen in Figure 5(c) depicting the dimerization ratio over exposure time, a rapid increase/decrease is observed in the initial stages of UV exposure before reaching a plateau, indicating both photodimerization and dedimerization of coumarin progress very effectively within a short period of time. Interestingly, the photolysis process is faster than photodimerization, because no diffusion of coumarin is needed. Figure 5(d) is the transmission electron microscope image of the polymer dispersions in a 0.1 wt % aqueous solution, which depicts spherical core–shell micellar morphology where the light-gray outer layer corresponds to the PEG chains, while the darker core is constructed from the peptide segments with higher electron density. Upon 365 nm UV irradiation for 2 h, coumarin dimerization induced significant aggregation within the micelle cores and altered the particle size and shape (Figure 5(e)). Changes in particle size are further analyzed using DLS illustrated in Figure 5(f) as dimerization leads to a slight reduction in particle size, while subsequent dedimerization with 254 nm UV light would partially recover its original dimension.

**Figure 5:**
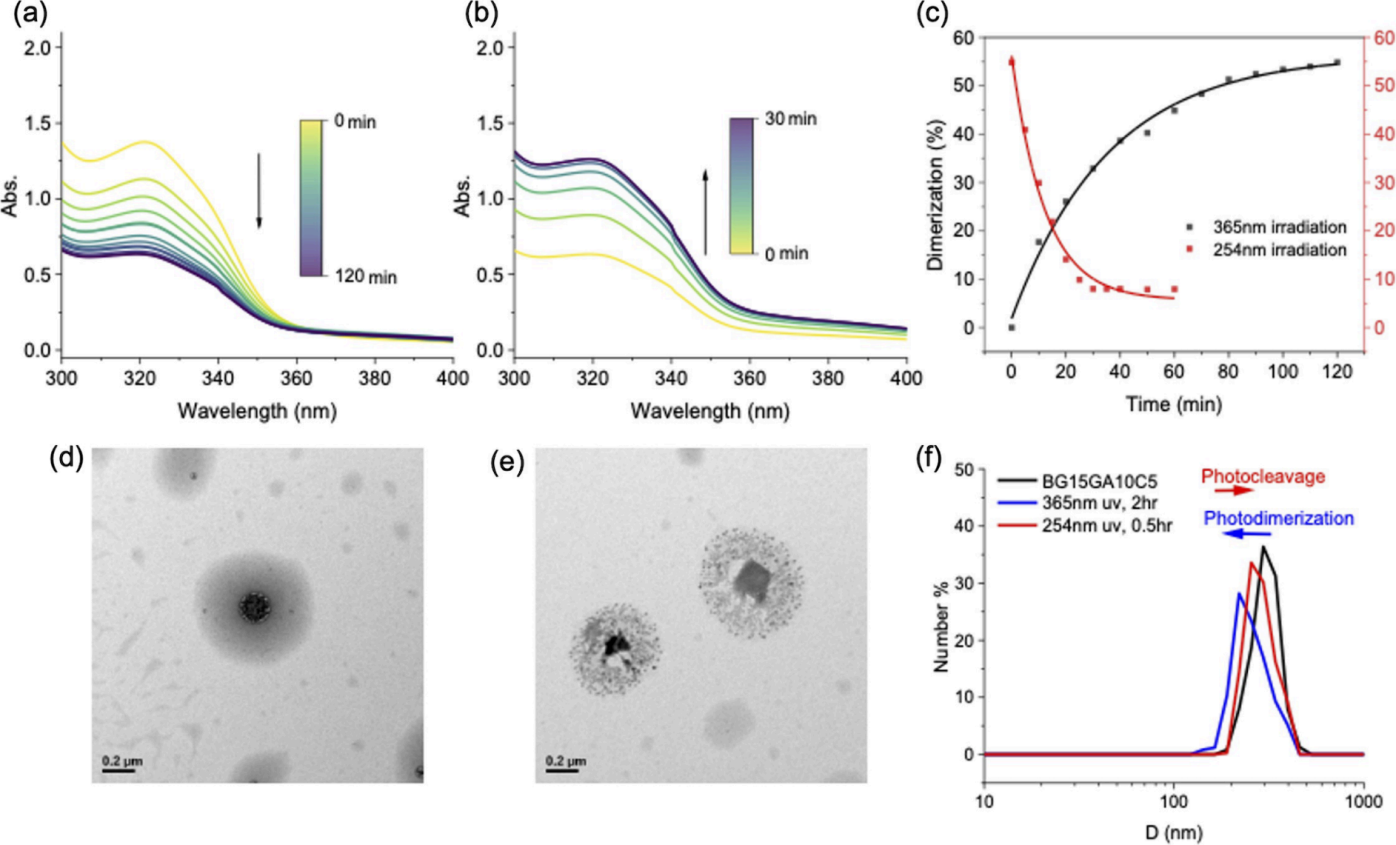
Time-dependent UV–vis absorption spectra of a 0.15 wt % BG15GA10C5 aqueous solution at (a) 365 nm UV irradiation; following by subsequent (b) 254 nm UV irradiation and (c) corresponding dimerization and dedimerization rates. TEM images of a 0.1 wt % BG15GA10C5 aqueous solution (d) before and (e) after 365 nm UV irradiation. (f) DLS analysis of particle size of the BG15GA10C5 solution upon photodimerization and photocleavage.

### Hydrogel Preparation and Mechanical Properties

Comprehensive investigations on the solubility of BG15GA10C5 in deionized water were conducted to ensure good solubility of the coumarin-functionalized PEG-peptide in water for successful hydrogel preparation. First, 5, 10, and 15 wt % aqueous solutions were prepared and subjected to 24 h ultrasonication to ensure complete dissolution. After standing for 72 h, slight scattering originating from micelles was observed for the 5 and 10 wt % solutions, while visible precipitation was clearly seen in the 15 wt % solution (Figure 6(c), red circle area). Therefore, the 10 wt % solution was selected to fabricate hydrogels because it should provide higher concentrations of BG units to promote nerve regeneration while retaining good solubility to facilitate hydrogel formation. As seen in Figure 6(d), the solution became more cloudy with white precipitates after 365 nm UV irradiation; and the white precipitates gradually disappeared upon subsequent 254 nm UV irradiation, indicating that the precipitates were photodimerized products. The observation suggests that BG15GA10C5 micelles only are unable to construct a stable cross-linked network in water for successful gelation even at high concentration.

**Figure 6:**
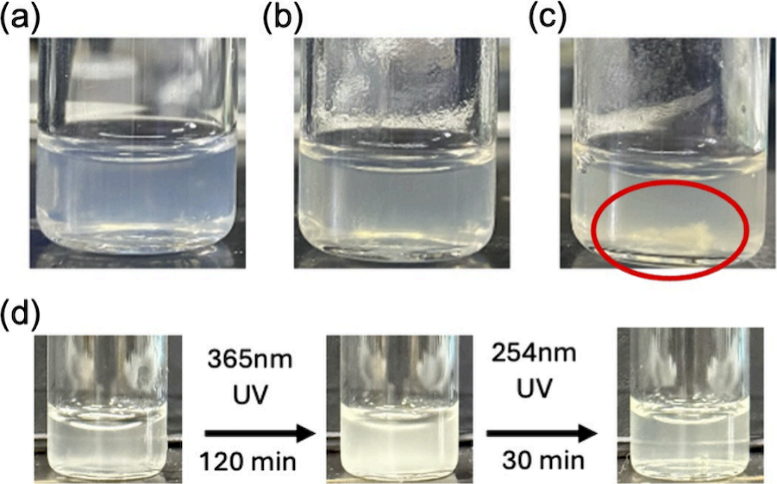
Photos of (a) 5 wt %, (b) 10 wt %, and (c) 15 wt % BG15GA10C5 aqueous solution. (d) The appearances of the 10 wt % BG15GA10C5 solution upon UV treatment for dimerization and dedimerization.

To achieve successful hydrogel production, we incorporated another water-soluble coumarin containing random copolymer poly­(*N*,*N*-dimethylacrylamide-*random*-acrylic­(7-(2-acryloyloxyethoxy)-4-methylcoumarin)) (PDA)^26^ to serve as “bridges” to connect the BG15GA10C5 core–shell micelles as schematically illustrated in Figure 7. PDA is a hydrophilic random copolymer that can dissolve well and extend in water, as confirmed by the transparent appearance of a 10 wt % PDA solution without noticeable scattering (Figure S13­(a)). Upon mixing PDA with BG15GA10C5, distinct scattering was observed to suggest the existence of BG15GA10C5 micelles, therefore supporting the presence of bridge-micelle structures in the mixture (Figures S13­(b) and S13­(c)). When the coumarin moieties of PDA approach those located within the cores of BG15GA10C5 micelles and undergo photodimerization, a cross-linked network is formed as PDA bridges interconnect BG15GA10C5 micelles (Figure 7).

**Figure 7:**
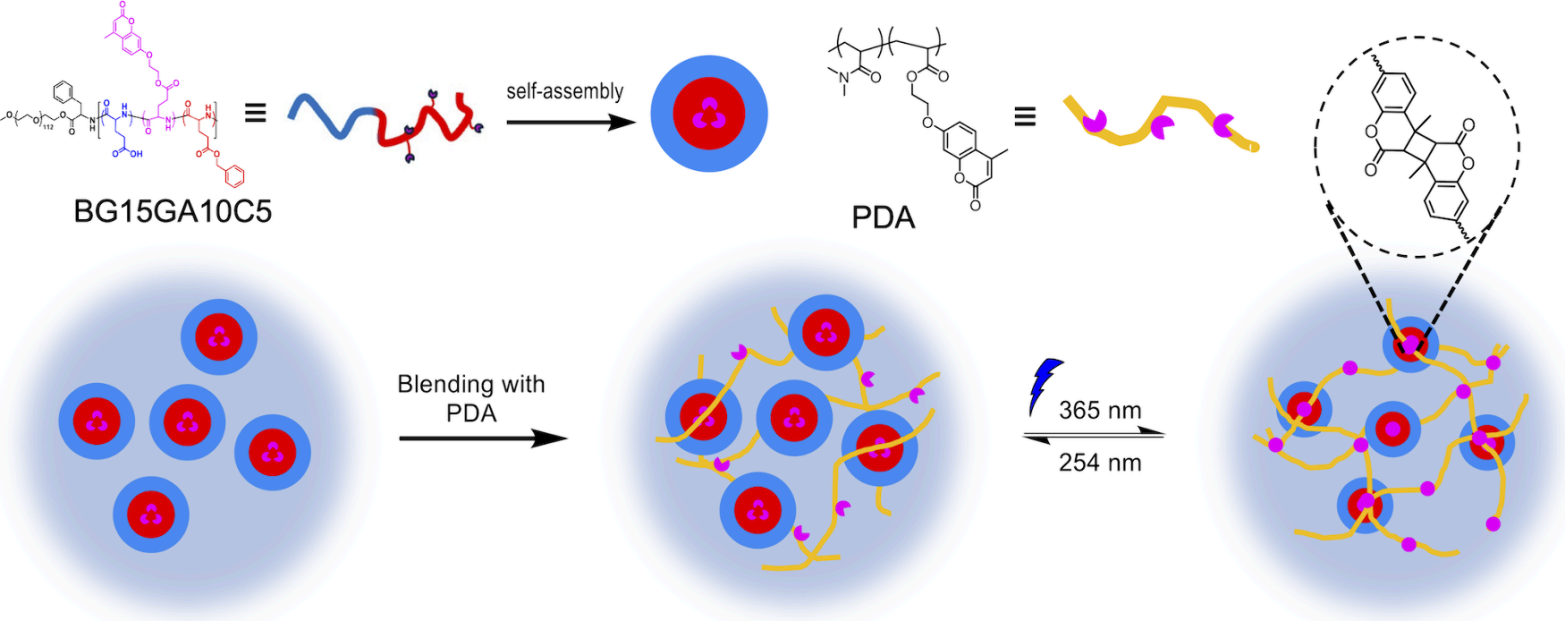
Schematic illustration of the PDA-BG15GA10C5 hydrogel formation mechanism.

Three hydrogel precursor solutions with different compositions were prepared and subjected to varied UV irradiation times to fabricate hydrogels. The precursor solutions are named as P*x*-PDA*y*, where *x* and *y* represent the weight percentage of BG15GA10C5 and PDA in the solution respectively; and the obtained hydrogels are termed P*x*-PDA*y*-T_1_, where T_1_ indicates the duration of 365 nm UV exposure in minutes. Neither P5 nor PDA5 containing only 5 wt % polymers can form hydrogels under prolonged UV exposure. When increasing the polymer concentration, PDA10, P5-PDA5, and P10-PDA5 exhibited homogeneous dispersions without precipitates (Figure S13), and all these three precursors could form hydrogels successfully after 30 min of 365 nm UV irradiation. To tailor the cross-linking density while preserving the structural integrity of the hydrogels, UV irradiation time was controlled between 0.5 and 2 h. Extended exposure beyond 2 h resulted in hydrogel rupture and mechanical failure, primarily attributed to excessive water evaporation during prolonged irradiation.

The mechanical properties and water absorption capability of hydrogels as biological scaffolds play critical roles for cell growth. The mechanical properties of the hydrogels are characterized by measuring the complex modulus (*G*) using a rheometer, and each measurement is repeated 3 times. The water absorption capability is closely related to the swelling ratio (SR), defined by the weight variation between the hydrogel and the corresponding dry samples, and a large swelling ratio generally refers to high water absorption capability. The SR of a hydrogel was calculated using eq 2, in which *W*_swollen_ refers to the weight of the water saturated hydrogel, while *W*_dry_ represents the weight of the water-free freeze-dried gel. Table 2 summarizes the complex modulus and swelling ratio of the hydrogels fabricated.

2 $$\mathrm{swelling ratio}\;\%=\frac{W_{\mathrm{swollen}}-W_{\mathrm{dry}}}{W_{\mathrm{dry}}}\times 100\%$$

**Table 2:** Complex Modulus and Swelling Ratio of the Hydrogels

sample	composition	365 nm UV irradiation time (min)	complex modulus (Pa)^a^	swelling ratio (%)
PDA10-30	10 wt % PDA	30	238 ± 43	1805 ± 171
PDA10-60		60	357 ± 41	1498 ± 118
PDA10-120		120	967 ± 39	912 ± 91
P5-PDA5-30	5 wt % PDA and 5 wt % BG15GA10C5	30	200 ± 26	1520 ± 114
P5-PDA5-60		60	311 ± 39	1390 ± 143
P5-PDA5-120		120	1163 ± 28	1089 ± 100
P10-PDA5-30	5 wt % PDA and 10 wt % BG15GA10C5	30	501 ± 49	1922 ± 154
P10-PDA5-60		60	689 ± 37	1680 ± 161
P10-PDA5-120		120	1448 ± 19	1180 ± 132

a The complex moduli were obtained from frequency sweep at 1.0 Hz.

As shown in Figure 8(a), within the linear viscoelastic range (defined as the strain range where the storage modulus is constant; see Figure S14), all the hydrogel systems exhibited significant increase in *G* with elongated UV exposure time, suggesting hydrogel stiffening due to increasing cross-linking density originated from the intensified dimerization reaction. Meanwhile, the increasing cross-linking density would concurrently suppress water absorption of the hydrogel as SR decreases with increasing irradiation time depicted in Figure 8(b). Interestingly, the variation of *G* and SR against UV exposure time of PDA10 and P5-PDA5 are different despite both of them having the same solid content of polymers and similar amount of coumarin groups in the hydrogels, which could be attributed to the difference in hydrogel architecture detailed as follows.

**Figure 8:**
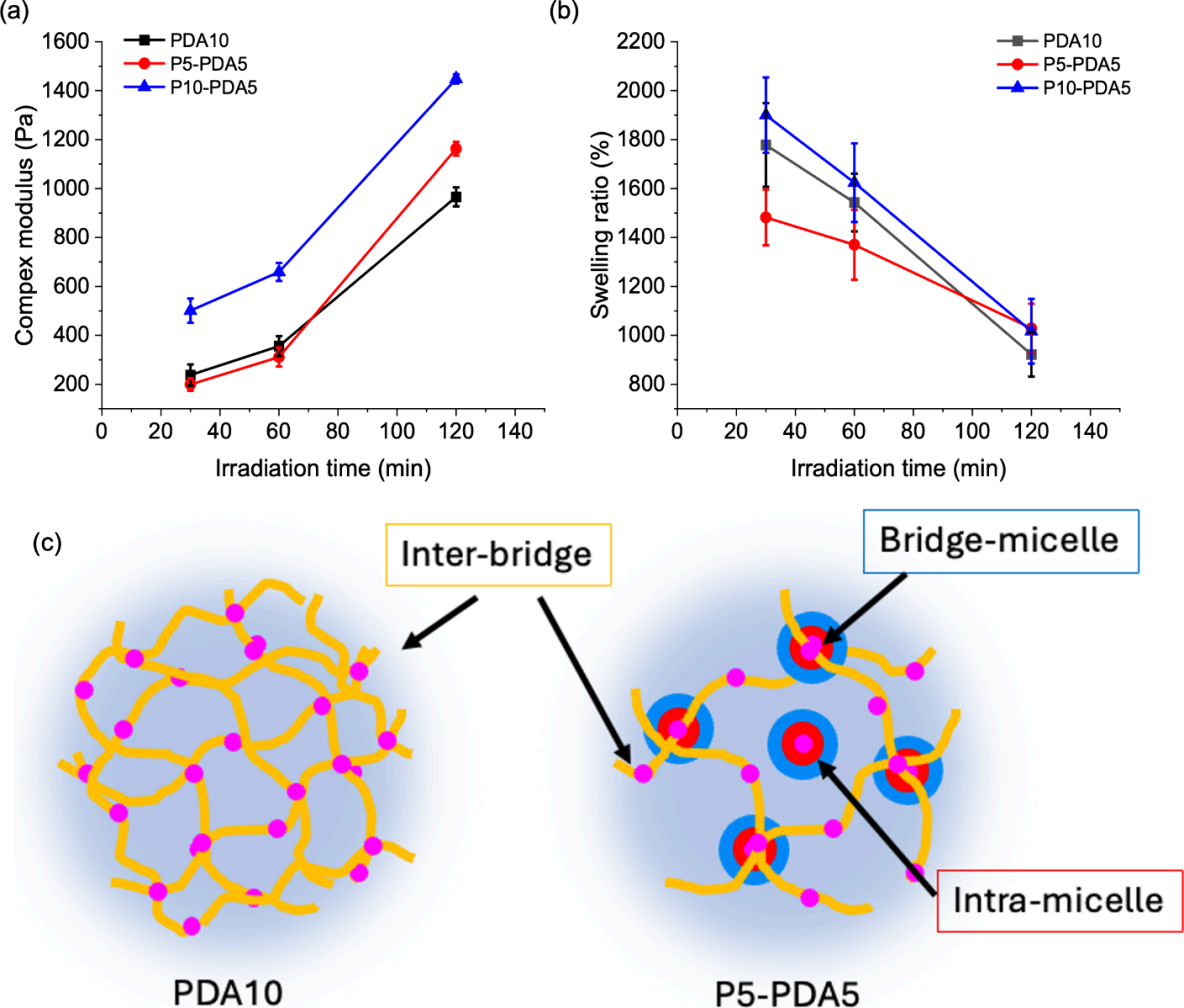
(a) Complex modulus and (b) swelling ratio of P*x*-PDA*y*-T_1_ hydrogels; (c) schematic illustration of three types of cross-linking in the hydrogels.

Three types of cross-linking are proposed in P5-PDA5, as illustrated in Figure 8(c). The first one is interbridge cross-linking between PDA chains, which should undergo predominately in the early stage of hydrogel formation because of free movement of extended PDA in the solution. The second type is bridge-micelle cross-linking between PDA chains and BG15GA10C5 micelles, which could occur in the early to middle stage to stabilize the hydrogel. The third type is intramacelle cross-linking within the core of BG15GA10C5 micelles, enabling the micelles to function as hardened nanoparticles to further enhance the mechanical strength. Meanwhile, for the PDA10 system, only interbridge cross-linking exists. As shown in Figure 8(a), when UV irradiation was applied for less than 60 min, the complex modulus of PDA10 is slightly higher than that of P5-PDA5 because of more interbridge cross-links in PDA10. However, after extending the irradiation time to 120 min, the complex modulus of P5-PDA5 surpassed PDA10, which could be attributed to the hardened micelles in P5-PDA5. It is also noticed that P5-PDA5-120 concurrently achieves SR higher than that of PDA10-120, even though the former has a higher complex modulus, which is contradictory to general observations suggesting that stiffer hydrogels usually absorb less water. Comparing to P5-PDA5-120, PDA10-120 has many more interbridge cross-links to limit the swelling of the hydrogel. Meanwhile, though hardened micelles in P5-PDA5-120 contribute to the improved mechanical strengths, they should not increase the number of cross-links outside the micelles so that more free volumes are available for water absorption.

The above discussions on the correlations between the hydrogel properties and the bridge-micelle architecture could be further validated by the behavior of P10-PDA5-T_1_ gels. Comparing P10-PDA5-T_1_ with P5-PDA5-T_1_, the former possesses double the concentration of BG15GA10C5 micelles but the same number of PDA chains. P10-PDA5-T_1_ exhibits a higher complex modulus throughout all UV irradiation times because of the contribution from more micelles. Notably, under identical UV exposure conditions, P10-PDA5-T_1_ exhibited a higher SR than P5-PDA5-T_1_, suggesting additional micelles should not increase swell-limiting cross-links. Moreover, the higher concentration of BG15GA10C5 micelles might simultaneously restrict the movement of PDA to decrease interbridge cross-linking and thus result in higher free volume for water absorption. The above observations highlights that the collaboration of bridge-micelle architectures and tailorable photo-cross-linking would enable hydrogels having a wide variety of mechanical and swelling properties without the need of tremendous works to build numerous polymers with different compositions.

Figure 9(a) reveals the mechanical properties of three highly cross-linked hydrogels, PDA10-120, P5-PDA5-120, and P10-PDA5-120, after exposure to 254 nm UV light for varied durations. All three hydrogels exhibited a gradual and consistent decrease in complex modulus with increasing irradiation time, suggesting dedimerization could perform effectively within a short period and the mechanical properties of a hydrogel could be dynamically modulated through the reversible light-responsivity of coumarin. Figure 9(b) shows the photos of P10-PDA5-120 hydrogel before and after 254 nm UV irradiation for 60 min. It is observed that after UV irradiation, part of the hydrogel became liquid due to insufficient cross-linking density to sustain stable network structure. The rheological results revealed in Figure S15 suggest the internal network becomes unstable after 30 min photocleavage, as the loss modulus surpasses the storage modulus with increasing angular frequency, indicating a transition toward liquid-like behavior. After 60 min of irradiation, the loss modulus is larger than the storage modulus throughout all the angular frequency, indicating liquid-like characteristics to confirm a complete gel–sol transition. This result indicates that reversible dimerization can be exploited as an effective strategy for clean removal of scaffolds without the need of additional reagents.

**Figure 9:**
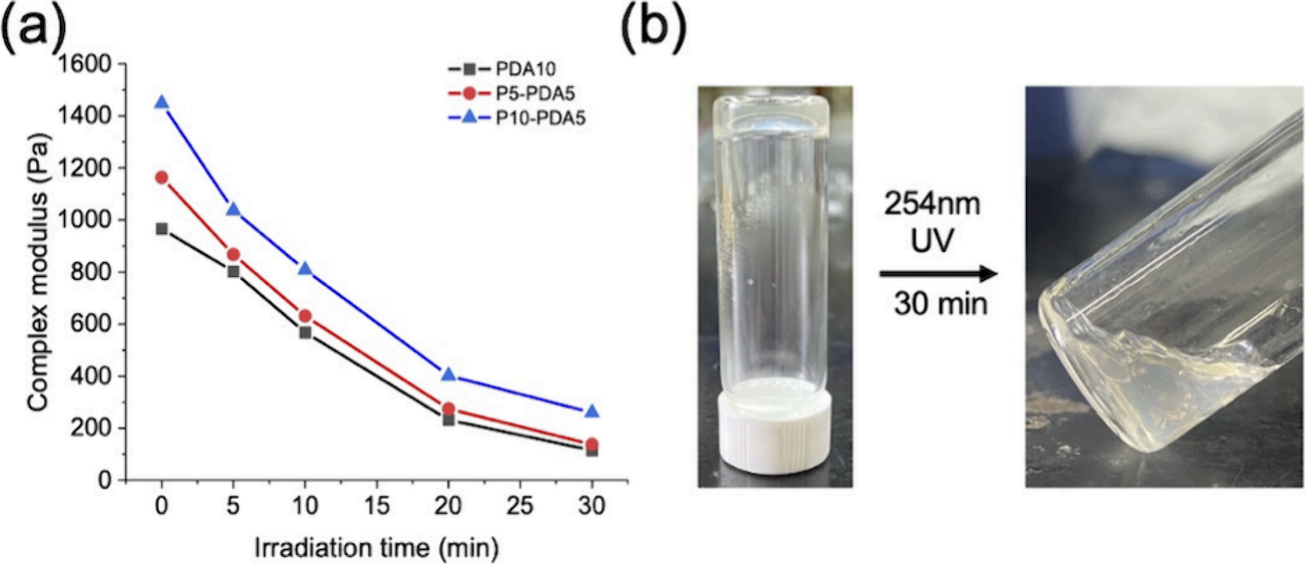
(a) Complex modulus of P*x*-PDA*y*-120 hydrogels after 254 nm UV light photolysis. (b) Photos of P10-PDA5-120 hydrogel before and after 254 nm UV photolysis for 30 min.

### Cell Behavior on Hydrogels

To assess the biocompatibility and cytotoxicity of the hydrogels, PC12 cells were adopted for in vitro cytotoxicity and cell viability tests. First, the hydrogels were fabricated on a tissue culture polystyrene (TCPS) well plate, cultured with PC12 for 7 days, and evaluated for biocompatibility using the live/dead assay. Cell cytotoxicity was observed using an optical microscope, as shown in Figure 10(a). The quantitative analysis was conducted by calculating the ratio of red (dead) to green (live) fluorescent areas, as shown in Figure 10(b). Compared with the lysine-coated control group with a cell survival rate of 57.3%, the hydrogels demonstrated higher cell survival rates, exceeding 75%. Notably, P5-PDA5-60, P5-PDA5-120, P5-PDA10-60, and P10-PDA5-120 showed over 95% cell viability, indicating that the improved mechanical strengths of the hydrogels significantly help cell survival.

**Figure 10:**
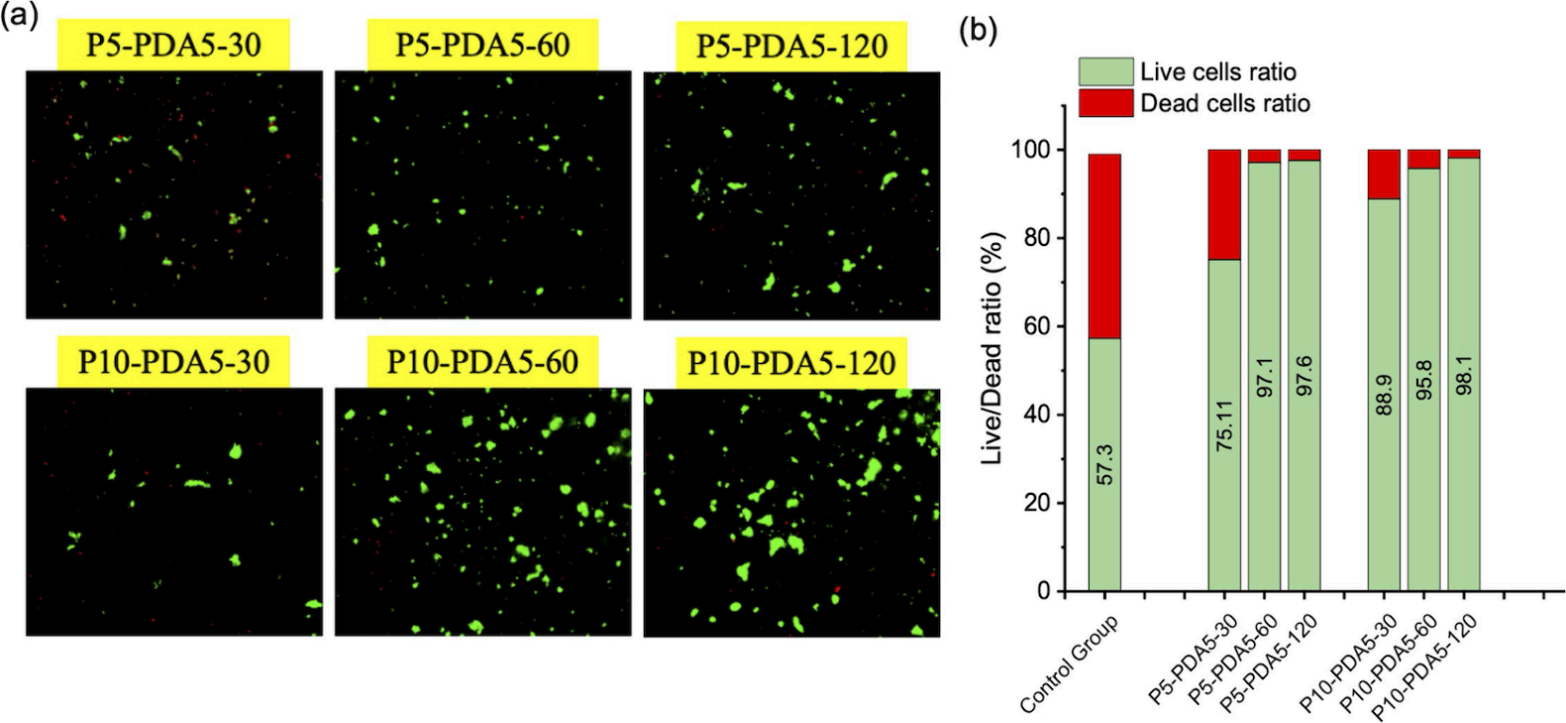
(a) Live/dead test fluorescent images and (b) live/dead ratios of PC12 cells using different hydrogels after 7 days of culture.

For the assessment of the cell viability, we utilized the Alamar Blue assay. The calculation details are provided in the Supporting Information (page 11). Figures 11(a) and 11(b) display cell proliferation at days 2, 4, and 7, all of which are normalized to the day 2 control group. On day 2, cell proliferation in all hydrogel systems exceeded that of the TCPS control group, indicating statistically significant differences. On day 4 and 7, the differences became even more pronounced (*p* < 0.005). Notably, in the P10-PDA5-120 hydrogel system, the cell viability of the cells reached 3.2 times to that of the control group. Although the increased viability of PC12 cells does not directly indicate nerve regeneration capability, the enhanced metabolic activity suggest that the hydrogels provide a supportive biochemical environment. Such an environment is generally favorable for subsequent PC12 differentiation toward a neuronal type in future studies.

**Figure 11:**
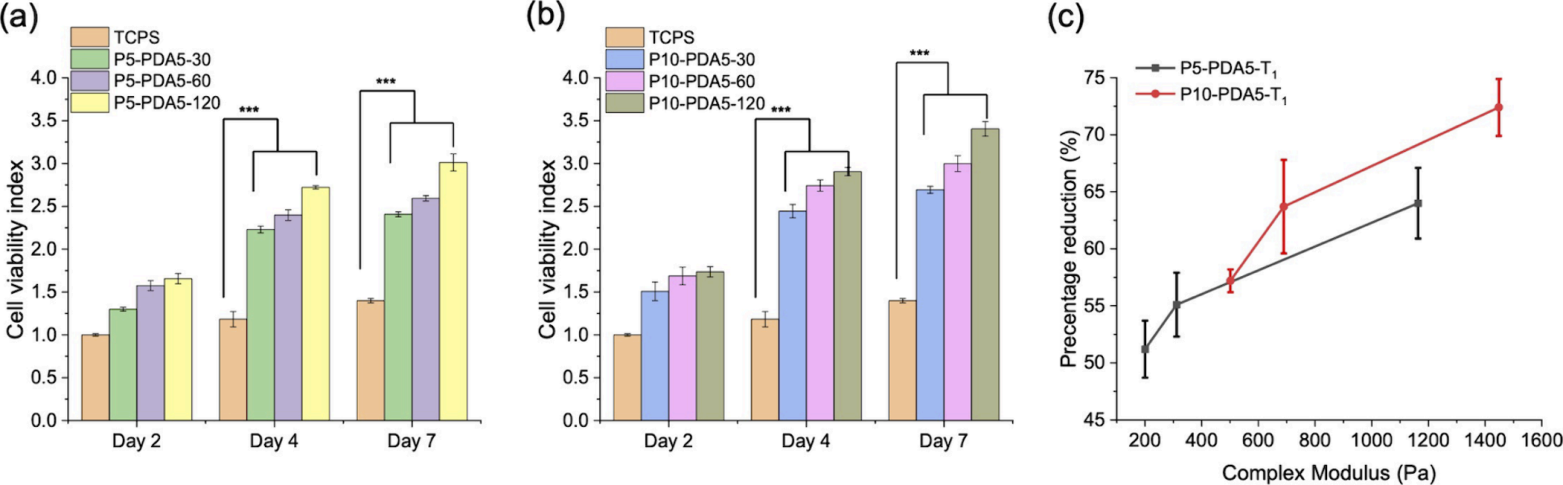
(a) Cell viability test of P5-PDA5-T_1_ series hydrogels on days 2, 4, and 7. (b) Cell viability test of P10-PDA5-T_1_ series hydrogels on days 2, 4, and 7. (c) Effect of mechanical strength on cell viability in hydrogel systems.

Comparing P5-PDA5-T_1_ with P10-PDA5-T_1_ hydrogels, PC12 cell growths reveal a positive correlation between PEG-peptide concentration and cell proliferation (Figure 11(c)). In P5-PDA5-T_1_ hydrogels, longer irradiation progressively enhanced the gel stiffness, which in turn promoted higher cell activity. A similar trend was evident in P10-PDA5-T_1_, confirming that increasing network rigidity is beneficial for supporting PC12 cell proliferation. Under identical UV irradiation conditions, P10-PDA5-T_1_ demonstrated a more substantial enhancement in PC12 cell growth compared to P5-PDA5-T_1_. While this improvement may be associated with the higher PBG content, the enhanced viability likely results from a combination of compositional and mechanical differences within the hydrogel formulations. Among all the hydrogels, P10-PDA5-T_1_ not only provided superior mechanical strength but also improved cell viability, suggesting a synergistic role of mechanical properties and biochemical cues in supporting cellular activity.

## Conclusions

In this study, we successfully synthesized a novel PEG-peptide block copolymer containing nerve cell growth stimuli, BG moieties, and light-responsive coumarin groups with controllable composition. By incorporating the PEG-peptide BCP and the coumarin containing random copolymer PDA, we could feasibly fabricate hydrogels through bridge-micelle network architectures for nerve tissue engineering applications. By adjustment of the UV exposure time at 365 nm and the composition of the precursor solution, the mechanical properties and the water absorption capability of the hydrogels could be feasibly modulated to optimize cell growth behaviors. Interestingly, it is observed that suitable UV exposure time would concurrently enhance the complex modulus and the swelling ratio of the hydrogel, and this unusual behavior could be explained by a proposed model based on the collaboration and competition of different cross-linking in the bridge-micelle network. PC12 cell growth studies show over 98% live/dead ratio, and the cell viability reaches 3.2 times that of the TCPS control group at best. It creates a supportive biochemical environment that benefits subsequent differentiation processes. Furthermore, the hydrogel could return to a solution upon 254 nm UV irradiation due to photodedimerization of coumarin, allowing clean removal of hydrogels. This hydrogel system provides a promising platform to construct photodegradable hydrogels for effective neurodegeneration, and a wide range of mechanical and swelling properties could be achieved without the need for tremendous works on adjusting the structure and the composition of constitutional polymers.

## Materials and Methods

### Materials

The following chemicals were purchased and used as received without further purification: poly­(ethylene glycol) monomethyl ether (mPEG, *M*_n_ = 5000, Aldrich), l-glutamic acid γ-benzyl ester (99% purity; Sigma), triphosgene (98% purity; Sigma), dichloroacetic acid (99% purity; Acros), 33 wt % HBr in acetic acid (99% purity; Acros), tetrahydrofuran (99% purity; THF; Sigma), d-DMSO (99.9%, Merck), d-chloroform (99.8%, Merck), ether (98% purity; Macron), hexane (95% purity; Unionward Corp., Taiwan), phosphate buffer saline (PBS; Sigma), dimethyl sulfoxide (99% purity; DMSO; Sigma), and dimethylformamide (99% purity; DMF, Acros). Syntheses of amino group terminated poly­(ethylene glycol) monomethyl ether (PEG-NH_2_), γ-benzyl-l-glutamate-*N*-carboxyanhydride (BG-NCA), 7-(2-hydroxyethoxy)-4-methyl-2H-chromen-2-one (HEOMC), 2-((4-methyl-2-oxo-2H-chromen-7-yl)­oxy)­ethyl acrylate (AC), and poly­(*N*,*N*-dimethylacrylamide-random-acrylic­(7-(2-acryloyloxyethoxy)-4-methylcoumarin)) (PDA) are detailed in the Supporting Information (pages 1–6). Chemicals for cellular behavior characterization include a live/dead viability/cytotoxicity kit (cat. no. L3224, Molecular Probes), Alamar Blue cell cytotoxicity assay (cat. no. BUF102A, AbD Serotec), and bovine serum albumin (BSA, cat. no. B4287, Sigma-Aldrich).

Instruments used for material characterization and cell culture observation included a nuclear magnetic resonance spectrometer (NMR; Bruker; HD-400), UV–vis spectrophotometer (V-650), dynamic light scattering analyzer (DLS, Zetasizer Nano), scanning electron microscope (SEM; JEOL; JSM-6700F), gel permeation chromatograph (GPC; Waters; DMF; Breeze 2), transmission electron microscope (TEM, JEOL, JEM2100F), rheometer (HR-2 system, TA Instruments), incubator (ESCO; 81022), laminar flow hood (ESCO; class II type A2), and optical microscope (DMI3000 B; Leica).

### Synthesis and Characterization of PEG-*b*-P­(BG-r-GA-r-C)

PEG-NH_2_ and BG-NCA were placed into two round-bottomed flasks separately in stoichiometric amounts. Both flasks underwent three freeze–thaw cycles to remove moisture and oxygen. Anhydrous DMF was then injected into the flasks using a syringe and the mixtures were stirred until appearing as clear solutions. The PEG-NH_2_ solution was then transferred into the BG-NCA solution, and the mixture was stirred under nitrogen at room temperature for 3 days. The reaction mixture was poured into diethyl ether to yield a white-yellow precipitate as crude PEG-*b*-PBG product. The crude product was redissolved in THF and reprecipitated into diethyl ether. This process was repeated three times to obtain PEG-*b*-PBG. PEG-*b*-PBG was then dissolved in dichloroacetic acid, following by the addition of designated amount of 33 wt % HBr in acetic acid. The reaction was proceeded at room temperature for 30 min to allow partial hydrolysis of PBG. The crude PEG-*b*-P­(BG-r-GA) product was precipitated from the resulting mixture by using diethyl ether. The crude product was redissolved in THF and subsequently precipitated into diethyl ether. This purification procedure was repeated three times to afford PEG-*b*-P­(BG-r-GA). The precipitate was then collected and dried under vacuum to a constant weight at room temperature.

PEG-*b*-P­(BG-r-GA) was dissolved in a DCM/DMF mixture (v/v = 3/1), and HEOMC was added for modification by using dicyclohexylcarbodiimide (DCC) and 4-*N*,*N*-dimethylaminopyridine (DMAP) as coupling reagents. The reaction is carried out at room temperature for 72 h. The solid byproduct, dicyclohexylurea (DCU), was removed by filtration, and the filtrate was poured into a large amount of diethyl ether to afford crude PEG-*b*-P­(BG-r-GA-r-C) precipitates. The precipitates were collected and dried under a vacuum to a constant weight at room temperature.

The chemical structures of the monomers and polymers were identified using nuclear magnetic resonance spectroscopy (NMR, Bruker HD-400). The molecular weight and polydispersity index of the polymers were obtained from GPC analysis using DMF as elutent with a flow rate of 1 mL min^–1^ and a calibration curve based on poly­(methyl methacrylate) (PMMA) standards. UV–vis spectroscopy was recorded from a 0.5 mg mL^–1^ aqueous solution upon exposing it to 365 or 254 nm UV light at a power of 20 mW cm^–2^.

### Characterization of Polymer Solutions

The morphologies of polymer dispersions in water were analyzed using JEOL JEM-2100F transmission electron microscopy (TEM). The 0.1 wt % aqueous solutions were dropped onto a carbon-coated copper grid, and water was removed using filter paper and air-dried at room temperature. The stability and particle size of the polymer dispersions in aqueous solution were characterized using the zeta potential and dynamic light scattering (DLS).

### Hydrogel Preparation and Characterization

The hydrogel precursor solutions were prepared by dissolving a designated amount of BG15GA10C5 and PDA in a phosphate-buffered saline (PBS) solution. To ensure complete dispersion, the mixture was sonicated prior to cross-linking. The solutions were then exposed to 365 nm UV light at 20 mW cm^–2^ for varying durations to form hydrogels. To rheological behaviors of a hydrogel at room temperature were evaluated using a rheometer (HR-2 system, TA Instruments) to record storage modulus (*G*′) and loss modulus (*G*″) as a function of time and frequency, as well as deformation through oscillatory measurements, as shown in the Supporting Information (Figure S14). The strain sweep was measured at a constant frequency of 10 rads/s over the strain range of 0.1–100% to determine the linear viscoelastic region (LVR) of the samples. During the frequency sweep tests, the shear rate was kept constant at strain = 0.1% and the frequency was changed between 0.1 and 100 Hz.

### Cell Culture

PC12 cells were cultured in RPMI-1640 medium supplemented with 10% (v/v) horse serum (HS), 5% (v/v) fetal bovine serum (FBS), and 1% (v/v) penicillin/streptomycin/amphotericin B (PSA). Cells were cultured at 37 °C under a humidified atmosphere containing 5% CO_2_. The culture medium was renewed every 3 days. The detailed cytotoxicity and cell viability calculations are shown in the Supporting Information. Cell viability was statistically analyzed using a two-tailed test, and all samples were measured in triplicate.

## Supplementary Material



## Notes

^1^H NMR characterization and procedures of monomers and polymer; hydrogel rheological measurements; images of mix hydrogel precursor solution; detailed cell experimental procedures (PDF)
