# Supplementary material for: A Nerve Cell Growth Promoting PEG-Peptide Block Copolymer and Photoresponsive Hydrogels with Tailorable Mechanical Properties and Feasible Degradability
Source: ACS Polym Au. 2026 Jan 20;6(1):415–25. doi: 10.1021/acspolymersau.5c00165 (PMC12903502; doi:10.1021/acspolymersau.5c00165)
Supplement: Supplementary file 1 [file lg5c00165_si_001.pdf]

Supporting Information

A Nerve Cell Growth Promoting PEG-peptide Block Copolymer and the Photo-responsive Hydrogels with Tailorable Mechanical Properties and Feasible Degradability

Syuan-Yu Lin[1], Wei-Fang Su[2], Chun-Yu Chang[3,4] and Chi-Yang Chao[1,5]**

[1]Department of Materials Science and Engineering, National Taiwan University, No. 1, Sec. 4, Roosevelt Road, Taipei 10617, Taiwan

[2]Department of Materials Engineering, Ming-Chi University of Technology, 84 Gungjuan Rd., Taishan Dist., New Taipei City 243303, Taiwan

[3]Bachelor Program in Semiconductor Materials and Fabrication, Ming Chi University of Technology, 84 Gungjuan Rd., Taishan Dist., New Taipei City 243303, Taiwan.

[4]Biochemical Technology R&D Center, Ming Chi University of Technology, 84 Gungjuan Rd., Taishan Dist., New Taipei City 243303, Taiwan

[5]Advanced Research Center for Green Materials Science and Technology, National Taiwan University, No. 1, Sec. 4, Roosevelt Road, Taipei 10617, Taiwan

Nomenclature

In this study, The PEG-peptide naming convention is BGpGAaCb. The γ-benzyl-L-glutamate segment is labeled as BG, L-glutamic acid as GA, and the GA units grafted with coumarin are denoted as C, with p, a, b are repeat unit numbers of BG, GA and C, respectively. In the hydrogel system, the hydrogel is composed of PEG-peptide and PDA. The naming convention is Px-PDAy-T_1, where P represents PEG-peptide, x is its weight percentage concentration. PDA refers to the blended polymer, y is its weight percentage concentration, T_1 indicates the 365nm UV light exposure time in minutes.

Synthesis of PEG-NH_2

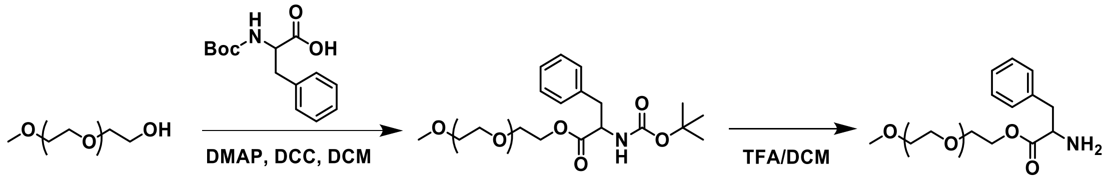

Figure S1. Synthetic scheme of PEG-NH_2.

N-(tert-Butoxycarbonyl)-L-phenylalanine (1 mmol), PEG (1 mmol) and DMAP (0.2 mmol) were dissolved in 50 mL dry dichloromethane (DCM) in a flame-dry flask, then DCC (2.0 mmol) dissolved in DCM was added slowly to the above solution with stirring and the reaction was conducted at room temperature for 48 h. The by-product dicyclohexylurea (DCU) precipitate was removed by filtration. Then, the solution was precipitated into excessive diethyl ether. The obtained product was further washed twice with diethyl ether and dried under vacuum at room temperature for 24 h (Yield: 96%). Subsequently, PEG-NH-t-Boc was dissolved in 20 mL DCM at 25 ^{o}C in a flask. After 20 mL trifluoroacetic acid (TFA) was added, the solution was slowly stirred at 25 ^{o}C for 2 h and then the final product was precipitated into excessive diethyl ether and washed twice with diethyl ether. The precipitate was collected and dried under vacuum to a constant weight at room temperature. The yield was approximately 88%. The ^{1}H NMR sprectra of PEG-NH-t-Boc and PEG-NH_2 are shown in **Figure S2**. Using the terminal methoxy protons of PEG (H_a) as the internal standard, the presence of 9 protons from the H_b Boc group was confirmed. After deprotection, the H_b signal

disappeared and a new H_c signal emerged, with the integral value in agreement with the expected number of protons, thereby confirming the successful deprotection.

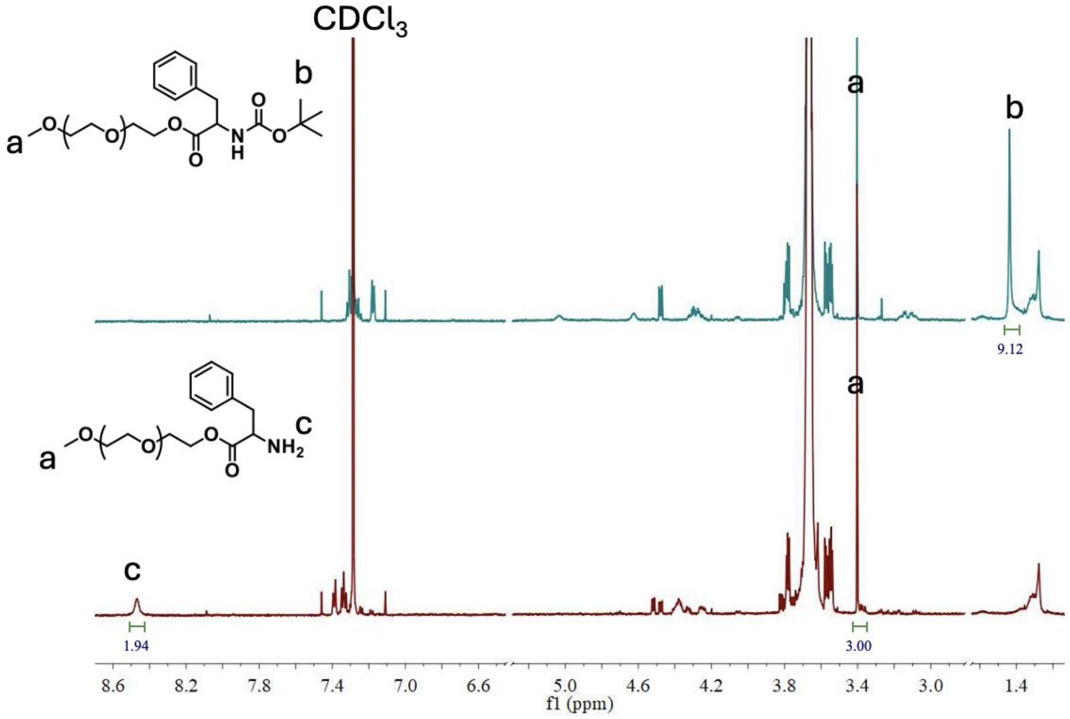

Figure S2. ^1H NMR spectra of PEG-NH_2.

Synthesis of γ-benzyl-L-glutamate-N-carboxyanhydride (BG-NCA)

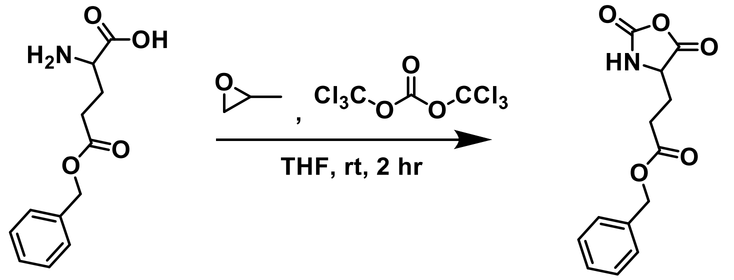

Figure S3. Synthetic scheme of BG-NCA.

To a single-neck 500 mL round flask, L-glutamic acid γ-benzyl ester (21.07mmol), anhydrous THF (50.0 mL), propylene oxide (85.8 mmol) were added sequentially with magnetic stirring. After 5 minutes of stirring utill the solution became homogeneous, triphosgene (12.13 mmol) was added quickly and the flask was sealed immediately. The mixture was stirred at room temperature for 5 hours. The product was precipitated

in hexane and vacuum-dried at 40°C overnight (92% yield). The ^{1}H NMR spectrum of BG-NCA is shown in **Figure S4.** ^{1}H NMR (600 MHz, CDCl3) δ 7.35 (ddd, J = 7.4, 5.2, 1.3 Hz, 5H), 6.60 (s, 1H), 5.14 (s, 2H), 4.39 – 4.35 (m, 1H), 2.59 (t, J = 6.9 Hz, 2H), 2.30 – 2.23 (m, 1H), 2.12 (dq, J = 14.2, 7.1 Hz, 1H).

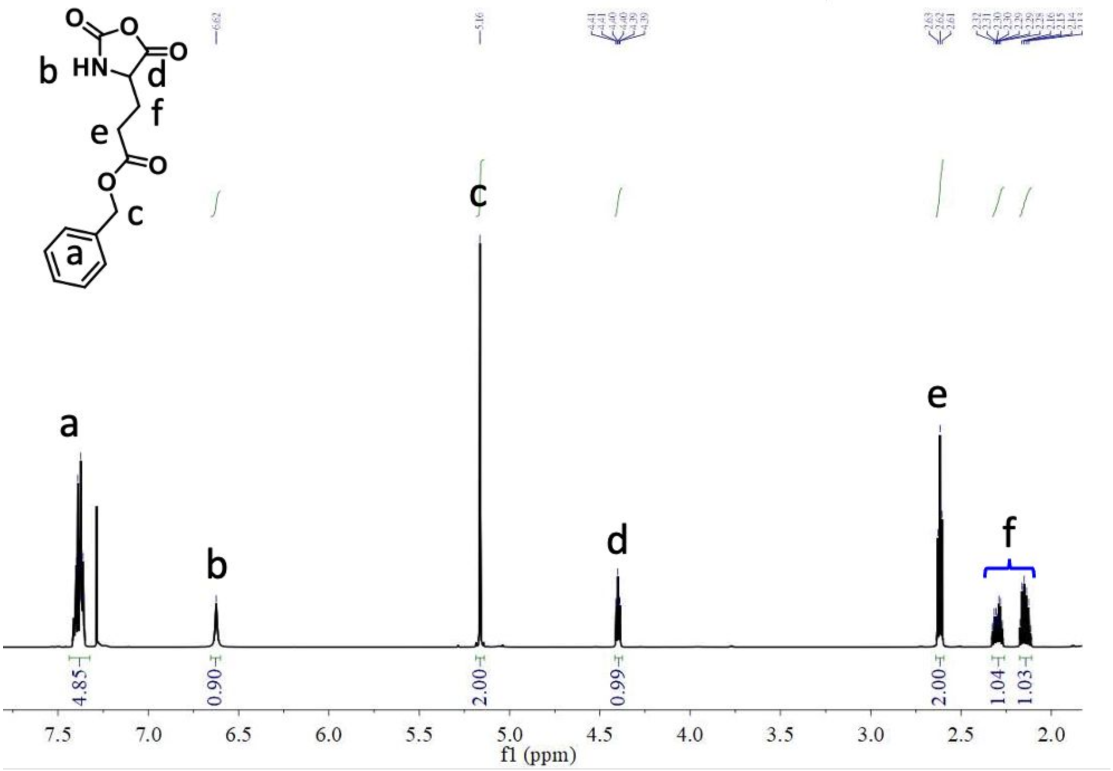

Figure S4. ^{1}H NMR spectra of BG-NCA.

Synthesis of 7-(2-hydroxyethoxy)-4-methylcoumarin (HEOMC)

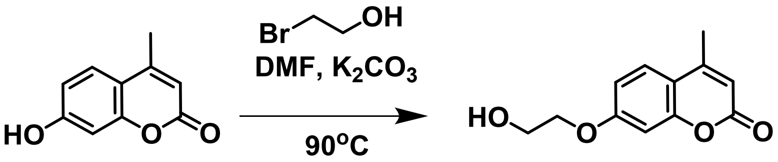

Figure S5. Synthetic scheme of HEOMC.

A mixture of 7-hydroxy-4-methylcoumarin (5.00 g, 28.38 mmol) and potassium carbonate (7.84 g, 56.76 mmol) in 50 mL of dry DMF was stirred for 15 min, and 2-bromoethanol (3.02 mL, 42.57 mmol) was added portion-wise to this solution. The reaction mixture was stirred for 18 h at 90 °C under a nitrogen atmosphere and then cooled down to room temperature. The mixture was poured into 200mL ice water, and

filtered to obtain the crude product as a white powder (96% yield). The ^{1}H NMR spectrum of HEOMC is shown in **Figure S6.** ^{1}H NMR (600 MHz, CDCl3) δ 7.50 (d, J = 8.8 Hz, 1H), 6.89 (d, J = 11.3 Hz, 1H), 6.83 (d, J = 2.5 Hz, 1H), 6.15 (s, 1H), 4.16 – 4.14 (m, 2H), 4.01 (d, J = 9.0 Hz, 2H), 2.40 (s, 3H).

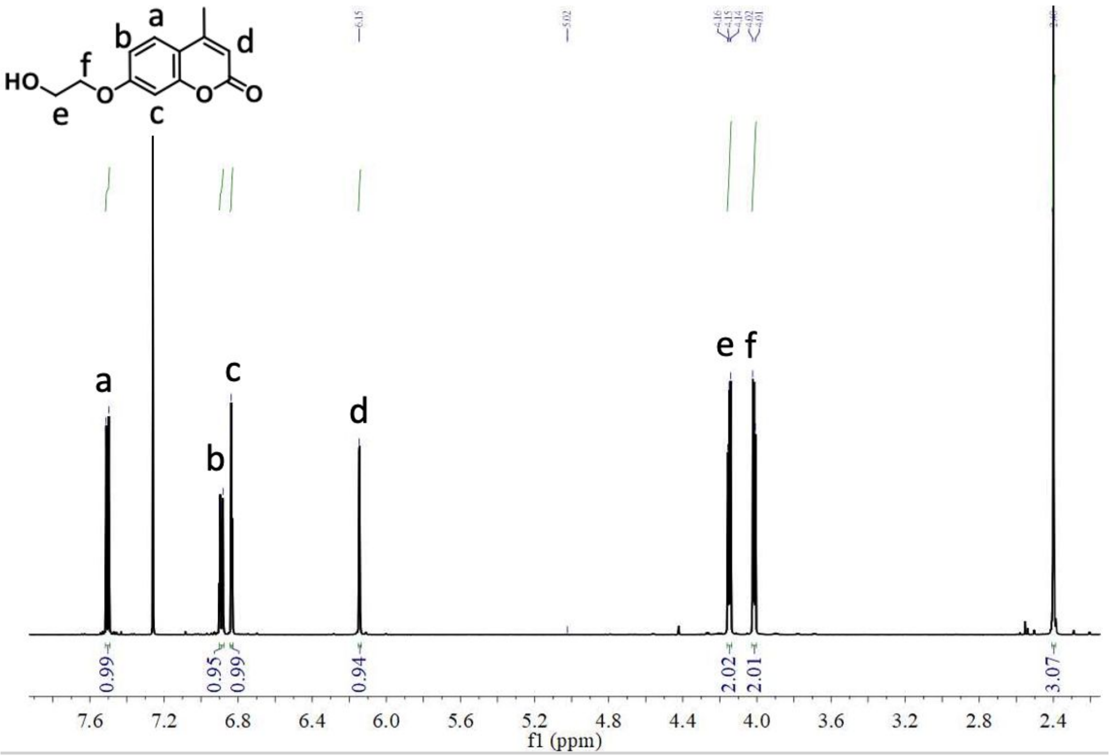

Figure S6. ^{1}H NMR spectra of HEOMC.

Synthesis of 7-acryloxyethoxy-4-methylcoumarin (AC)

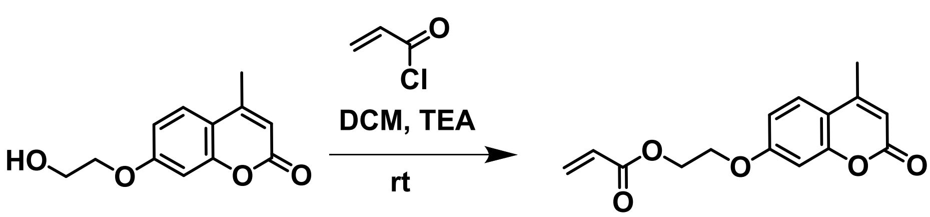

Figure S7. Synthetic scheme of AC

Triethylamine (2.2 g, 2.5 mL, 1.57 mmol) and HEOMC (3.0 g, 13.62 mmol) were dissolved in 80 mL of dichloromethane, and into which acryloyl chloride (2.80 g, 3 mL, 30.936 mmol) was added dropwisely. After stirring for 12 h at room temperature, 100

mL brine was added to quench excess acryloyl chloride and extraction. The DCM layer was collected, and the aqueous layer was extracted with dichloromethane. Followed by drying over anhydrous sodium sulfate and the removal of the solvent to give a solid product (77% yield). The 1H NMR spectrum of AC is shown in **Figure S8.** ^{1}H NMR (600 MHz, CDCl3): δ (ppm) 7.51 (d, 1H), 6.89 (dd, 1H), 6.82 (d, 1H), 6.48-6.44 (dd, 1H), 6.20-6.14 (m, 2H), 5.89-5.86 (dd, 1H), 4.55 (t, 2H), 4.28 (t, 2H), 2.39 (s, 3H).

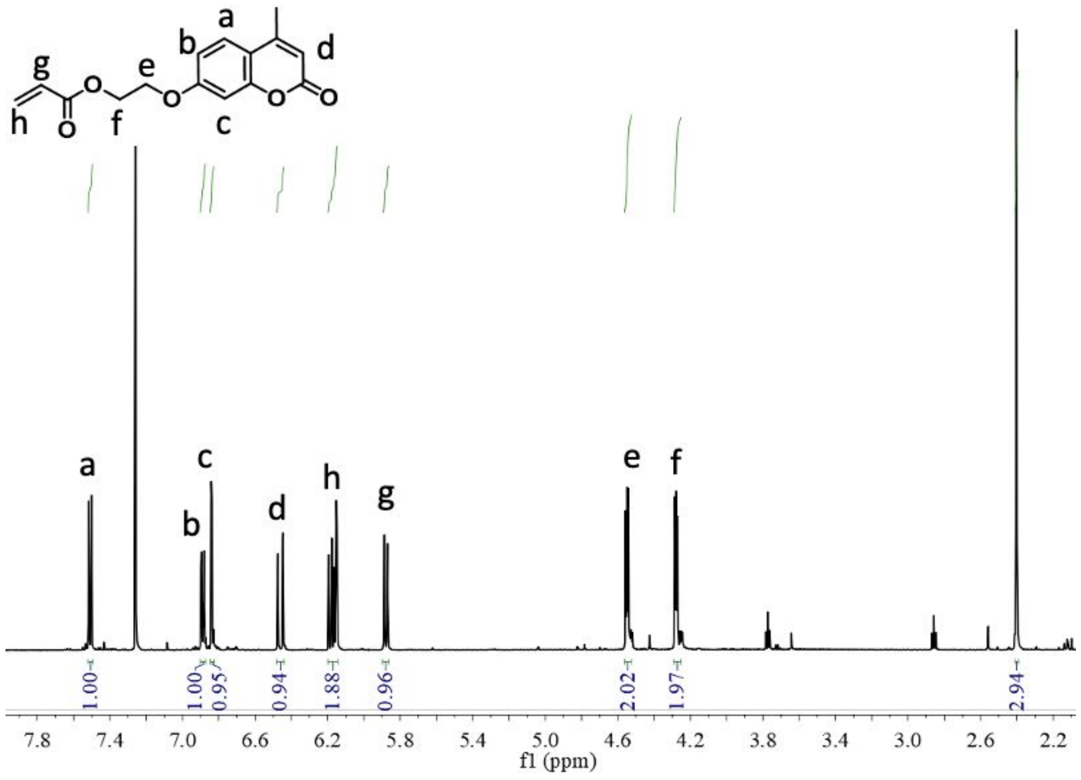

Figure S8. ^{1}H NMR spectra of AC.

Synthesis of PDA

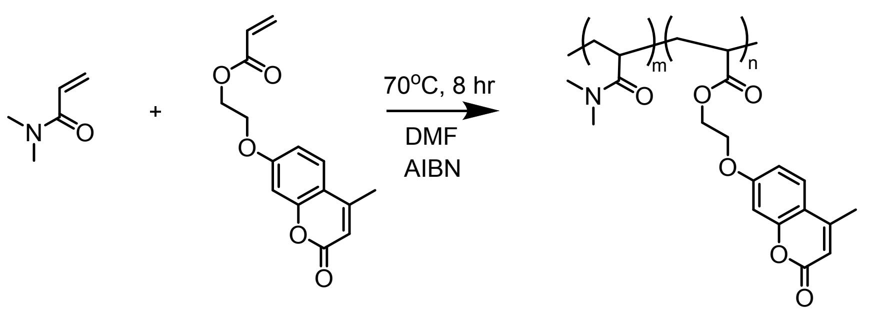

Figure S9. Synthetic scheme of PDA.

In a typical copolymerization, DMA (500 mg, 5.04 mmol), AC (72.8 mg, 0.266 mmol), and AIBN (17.4 mg, 0.106 mmol, 0.02 equiv. relative to total monomer) were dissolved in DMF in a 100 mL round bottom flask and purged with nitrogen. The flask was placed in a preheated heating block at 60 °C and the polymerization was allowed to perform for 8 h. The reaction mixture was precipitated in ether to yield the crude copolymer. The crude product was redissolved in THF and reprecipitated into diethyl ether. This process was repeated three times to obtain PDA. The ^1H NMR spectrum of PDA is shown in **Figure S10.** In the random copolymer PDA, the composition ratio of AC was denoted as n and DMA as m. The monomer ratio was calculated based on the integration values of characteristic peaks. Specifically, H_a, H_b, and H_c correspond to AC, while H_d corresponds to DMA. By setting the integration value of H_a to 1, representing one aromatic proton of AC, and considering H_c with an integration value of 68.39 corresponding to six protons of DMA, the ratio was determined as 1/1 : 68.39/6 ≈ 1 : 20. Therefore, n = 1 and m = 20.

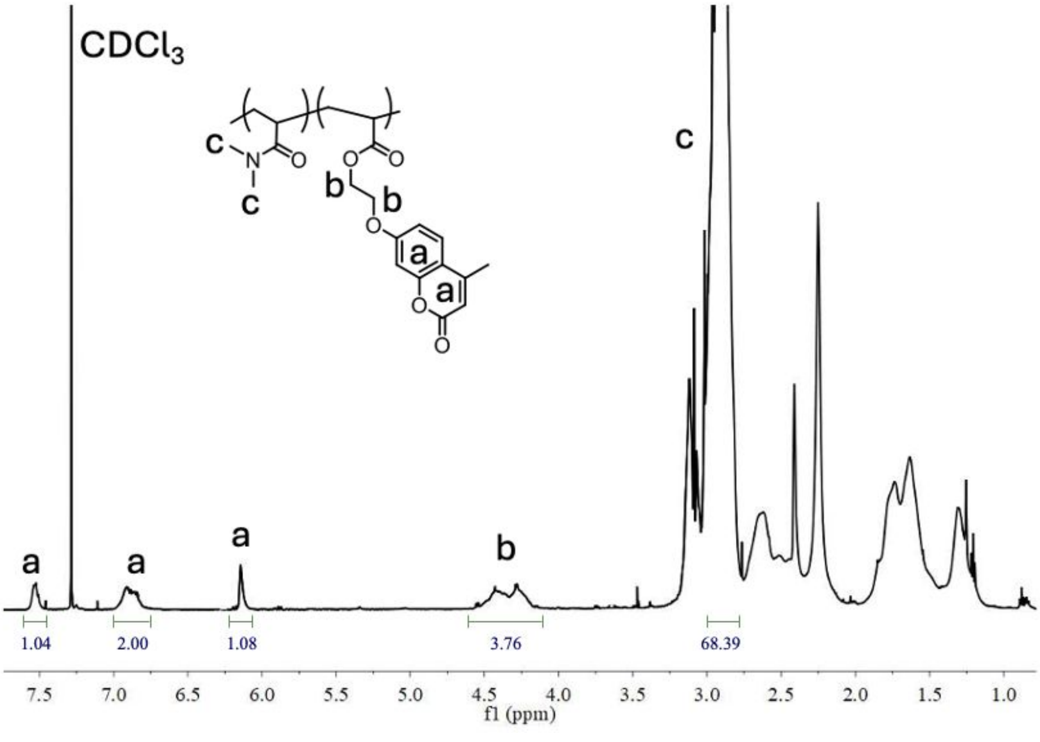

Figure S10. ^1H NMR spectra of PDA.

Composition of PEG-b-P(BG-r-GA) evaluated by ^{1}H NMR spectra

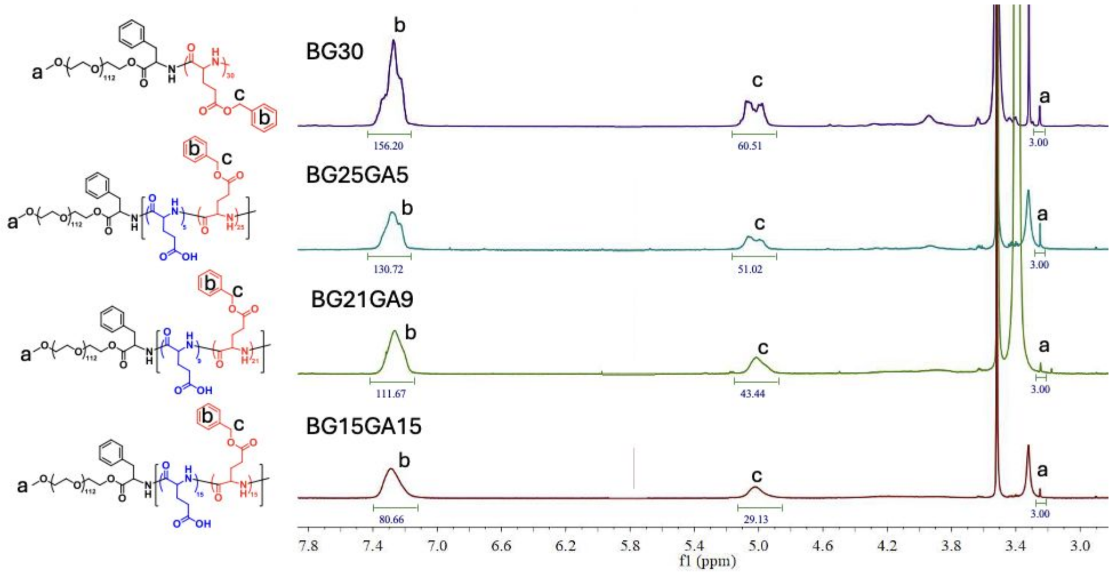

Figure S11. ^{1}H NMR spectra of PEG-b-PBG and PEG-b-P(BG-r-GA)

Figure S11 shows the ^{1}H NMR spectra of PEG-b-PBG and PEG-b-P(BG-r-GA) series including BG30, BG25GA5, BG21GA9 and BG15GA15. The integral of H_a at $\delta = 3.26$ ppm was set to 3, corresponding to the three terminal methoxy protons of PEG. Take BG30 for example, the integral value of H_b at $\delta = 7.2$–7.42 ppm corresponding to the aromatic protons from PBG was determined to be 156.20. However, the five aromatic protons contributed by the PEG-NH_2 end group need to be subtracted, and the adjusted value becomes (156.20 – 5) = 151.20. Since each BG unit contains five aromatic protons, dividing 151.20 by 5 gives approximately 30 BG units. An alternative and more intuitive calculation method using the integral value of H_c at $\delta = 4.85$–5.15 ppm, which corresponds to the two protons of the BG benzyl group. In BG30, the integral value at this region is 60.51. By dividing 60.51 by 2, we obtain approximately 30 BG units, confirming the polymer composition. The same methodology was applied to calculate the number of BG units (p) in BGpGAq. Since the total number of BG units and GA units in BGpGAq should equal to the number of BG units in BGn, i.e. $p + q = 30$, we can have q accordingly.

Composition of PEG-b-P(BG-r-GA-r-C) calculated from ^{1}H NMR spectrum

The integral at δ = 3.26 ppm was set to 3, corresponding to the three terminal methoxy protons of PEG. The integral values of H_d at δ = 6.2–6.4 ppm and H_b δ = 7.5–7.7 ppm were 5.12 and 4.87 respectively resulting in approximate normalized values of 5 (5.12/1 ≈ 5 and 4.87/1 ≈ 5). Additionally, the integral of H_c at δ = 6.75–7.1 ppm was 10.54. Dividing this value by two yields approximately 5.27 (10.54/2 ≈ 5.27), which aligns with the previous normalized values. By using these integrals and considering the known proton contributions from each HEMOC unit, the number of coumarin groups grafted onto the copolymer was calculated to be approximately five per chain, suggesting b = 5 in BGpGAaCb and a = 10 as a + b = 15.

In PEG-b-P(BG-r-GA-r-C), the expected molecular weight of PEG-NH2 is 5147 Da, while the molecular weights of the repeating units are 236 Da for BG, 146 Da for GA, and 220 Da for HEOMC. Based on the NMR-determined composition of BG15GA10C5, the weight fraction of C in the structure was calculated as follows: (220*5)/(5147+236*15+146*15+220*5) *100% =9.2%

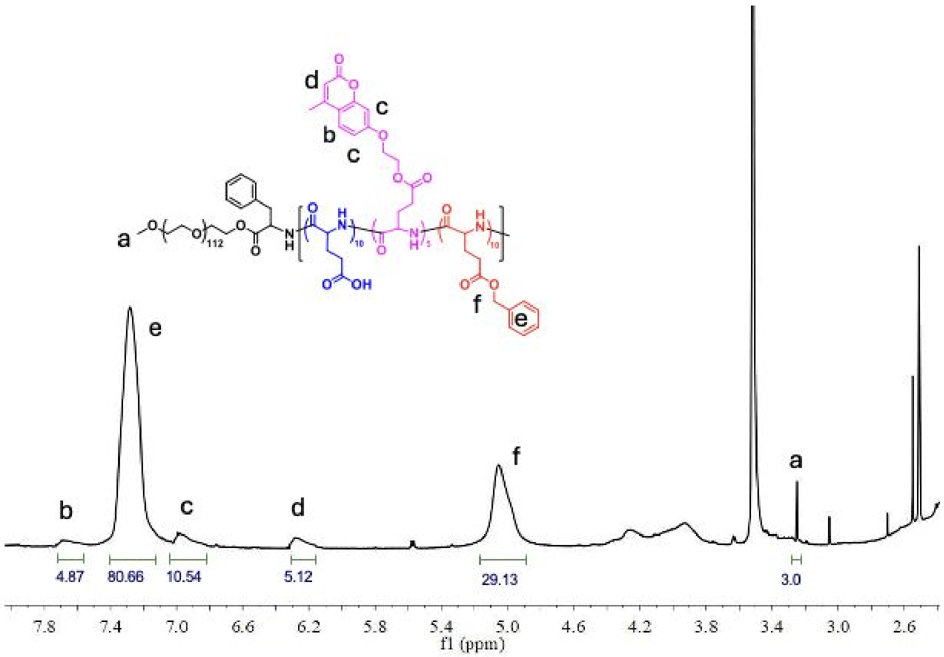

Figure S12. ^{1}H NMR spectra of BG15GA10C5

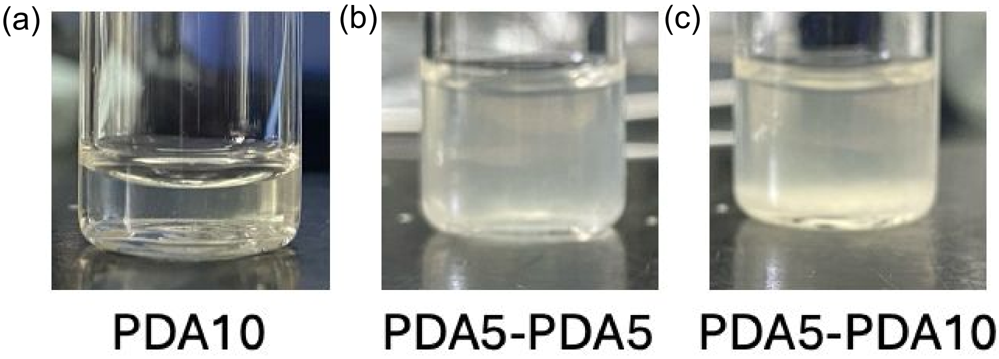

Figure S13. Photos of (a) PDA10, (b) P5-PDA5 and (c) P10-PDA5 aqueous solution.

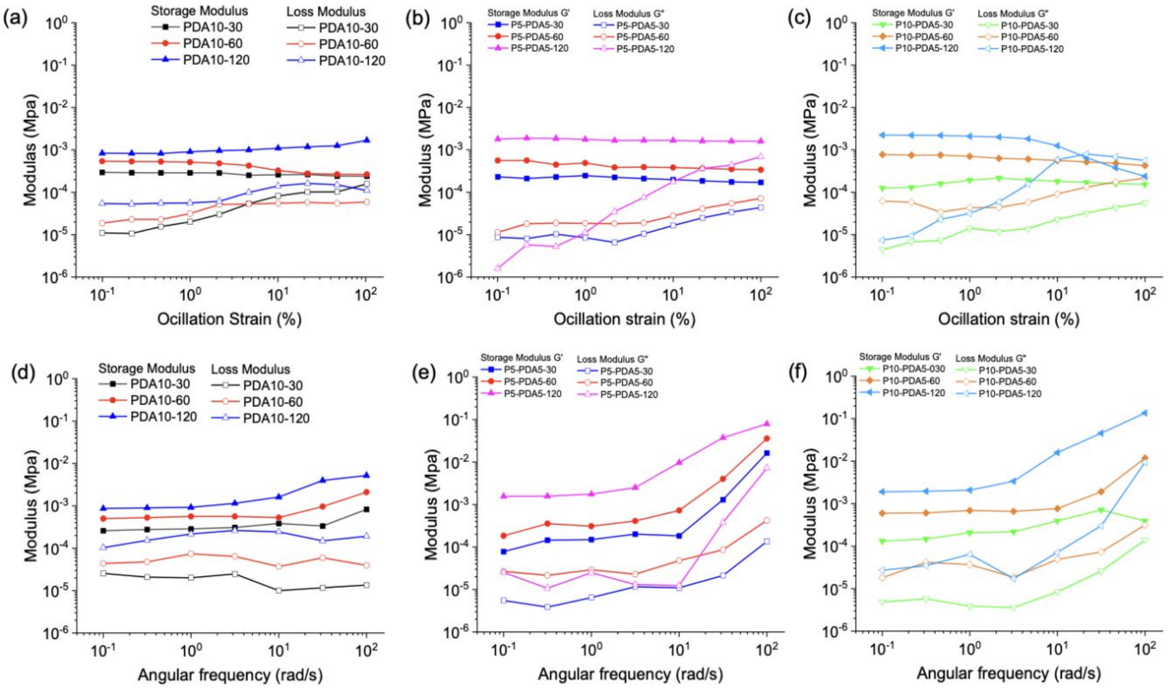

Figure S14. Strain sweep results for (a)P10-T_1, (b)P5-PDA5-T_1 and (c)P10-PDA5-T_1 hydrogels. Frequency sweep results for (d)P10-T_1, (e)P5-PDA5-T_1 and (f)P10-PDA5-T_1 hydrogels.

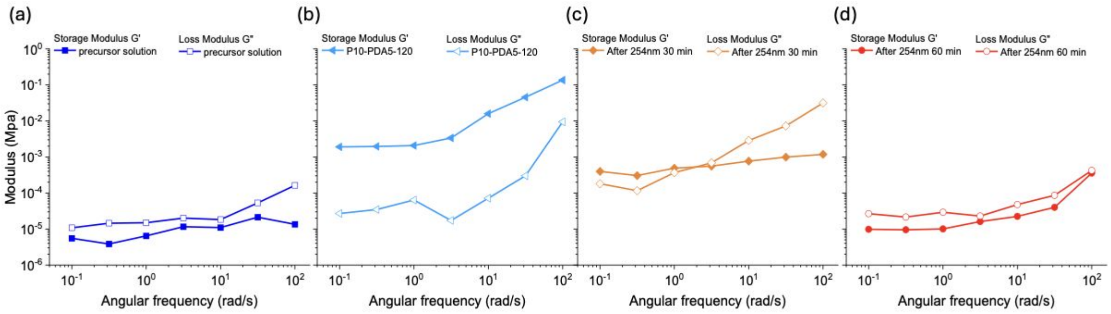

Figure S15. Frequency sweep results for P10-PDA5-120 hydrogel at each status including (a)precursor solution, (b) gel states and after 254nm UV induced dedimerization for (c) 30 and (d) 60 minutes.

Live/dead assay

Scaffolds on cover glasses were exposed to UV light for 2 hours and then immersed in fresh culture medium for 20 minutes until before use. PC12 cells were seeded on scaffolds in a density of 10000 cells/cm2. After 7 days, cells were stained with 0.05% (v/v in PBS) calcein-AM for live cells and 0.2% (v/v in PBS) ethidium homodimer-1 (EthD-1) for dead cells at room temperature for 2 hours. The samples were washed with PBS and ready for fluorescence optical microscopy.

Alamar Blue assay.

Hydrogels were prepared and PC12 cells were seeded in a same way as the way adopted in the live/dead assay. After 2 day (*i.e.*, on Day 2), the culture medium was replaced with the diluted Alamar Blue solution (10% v/v in DMEM high glucose without phenol red). After 4 hours of incubation at 37℃, 5% CO_2, the solution was carefully pipetting and taken out to another 96-well plate to test the absorption at 570 and 600 nm. The dye reduction percentage was calculated by **equation (3)**. At the same time, cells were added with fresh medium and put back to incubator to continue the cell culture. On Day 4 and 6, the procedure was repeated.

$$\text{percent reduced (\%)} = \frac{\varepsilon_{ox}\lambda_{600} A_{\lambda 570} - \varepsilon_{ox}\lambda_{570} A_{\lambda 600}}{\varepsilon_{red}\lambda_{570} A'_{\lambda 600} - \varepsilon_{red}\lambda_{600} A'_{\lambda 570}} \qquad \textbf{\textit{eq}(3)}$$

$\varepsilon_{ox}\lambda_{570}$=80586 ; $\varepsilon_{red}\lambda_{570}$= 155677, $\varepsilon_{ox}\lambda_{600}$=117216 ; $\varepsilon_{red}\lambda_{600}$= 14652

A: Absorption of test sample; A’: Absorption of blank
